# Integrative Transcriptomic Analysis Reveals the Immune Mechanism for a CyHV-3-Resistant Common Carp Strain

**DOI:** 10.3389/fimmu.2021.687151

**Published:** 2021-07-05

**Authors:** Zhiying Jia, Nan Wu, Xiaona Jiang, Heng Li, Jiaxin Sun, Mijuan Shi, Chitao Li, Yanlong Ge, Xuesong Hu, Weidong Ye, Ying Tang, Junwei Shan, Yingyin Cheng, Xiao-Qin Xia, Lianyu Shi

**Affiliations:** ^1^ Heilongjiang River Fisheries Research Institute, Chinese Academy of Fishery Sciences, Harbin, China; ^2^ National and Local Joint Engineering Laboratory for Freshwater Fish Breeding, Harbin, China; ^3^ Key Laboratory of Aquatic Genomics, Ministry of Agriculture, Chinese Academy of Fishery Sciences, Beijing, China; ^4^ Institute of Hydrobiology, Chinese Academy of Sciences, Wuhan, China; ^5^ College of Advanced Agricultural Sciences, University of Chinese Academy of Sciences, Beijing, China; ^6^ College of Fisheries and Life Science, Dalian Ocean University, Dalian, China; ^7^ The Innovative Academy of Seed Design, Chinese Academy of Sciences, Beijing, China

**Keywords:** transcriptome, lncRNA, CyHV-3, resistant immune mechanism, common carp

## Abstract

Anti-disease breeding is becoming the most promising solution to cyprinid herpesvirus-3 (CyHV-3) infection, the major threat to common carp aquaculture. Virus challenging studies suggested that a breeding strain of common carp developed resistance to CyHV-3 infection. This study illustrates the immune mechanisms involved in both sensitivity and anti-virus ability for CyHV3 infection in fish. An integrative analysis of the protein-coding genes and long non-coding RNAs (lncRNAs) using transcriptomic data was performed. Tissues from the head kidney of common carp were extracted at days 0 (the healthy control) and 7 after CyHV-3 infection (the survivors) and used to analyze the transcriptome through both Illumina and PacBio sequencing. Following analysis of the GO terms and KEGG pathways involved, the immune-related terms and pathways were merged. To dig out details on the immune aspect, the DEGs were filtered using the current common carp immune gene library. Immune gene categories and their corresponding genes in different comparison groups were revealed. Also, the immunological Gene Ontology terms for lncRNA modulation were retained. The weighted gene co-expression network analysis was used to reveal the regulation of immune genes by lncRNA. The results demonstrated that the breeding carp strain develops a marked resistance to CyHV-3 infection through a specific innate immune mechanism. The featured biological processes were autophagy, phagocytosis, cytotoxicity, and virus blockage by lectins and MUC3. Moreover, the immune-suppressive signals, such as suppression of IL21R on STAT3, PI3K mediated inhibition of inflammation by dopamine upon infection, as well as the inhibition of NLRC3 on STING during a steady state. Possible susceptible factors for CyHV-3, such as ITGB1, TLR18, and CCL4, were also revealed from the non-breeding strain. The results of this study also suggested that Nramp and PAI regulated by LncRNA could facilitate virus infection and proliferation for infected cells respectively, while T cell leukemia homeobox 3 (TLX3), as well as galectin 3 function by lncRNA, may play a role in the resistance mechanism. Therefore, immune factors that are immunogenetically insensitive or susceptible to CyHV-3 infection have been revealed.

## Introduction

Cyprinid herpesvirus-3 (CyHV-3) infection is a major threat to common carp aquaculture (1), leading to widespread mortality and substantial economic loss. CyHV-3 is thought to cause death by weakening the host’s immune system, resulting in susceptibility to pathogenic microbes (1). In common carp, clinical signs of the disease develop rapidly and may induce morbidity and mortality within a period of 6 to 10 days following infection (2).

Carp that survive a primary infection with CyHV-3 can be resistant to future infection with this virus. Since latency and persistent carrying of CyHV-3 exist in carp (2–4), genetic backgrounds are crucial in developing an understanding of resistance against the virus. Experimental infections of carp from pure lines or crosses have indicated the existence of a genetic background of resistance by divergent survival rates (2). For example, a markedly higher expression of immune-related genes involved in pathogen recognition, complement activation, major histocompatibility complex class I (MHC I)-restricted antigen presentation, and the development of adaptive mucosal immunity was noted in the more resistant R3 line. Higher activation of CD8^+^ T cells was also observed (5). The diallelic cross of four European carp lines, including Polish ‘K’ and ‘R6’, Hungarian ‘R7’ and French ‘F’ also has been done to select the resistant fish, and then found that MH class II B genes of carp can affect immunity against CyHV-3 infection (6). Additionally, carp strains of Asian origin, particularly Amur wild carp, were shown to be more resistant to CyHV-3 than strains originating from Europe, such as the Prerov scale carp or koi carp from a breed in the Czech Republic (7).

The immune response of carp to CyHV-3 involves both innate and adaptive aspects with the outcome of the disease largely depending on whether the balance is tipped in favor of the host’s immune response or virus’s evasion strategy (2). In general, transcriptomic analysis has revealed that three immune-related pathways, namely the mitogen-activated protein kinase (MAPK) signaling pathway, the innate immune response, and the cytokine-mediated signaling pathway, were highly involved in the infection with CyHV-3 (8). In red common carp × koi, the expression of interleukin 12 (IL12) p35, interferon (IFN) αβ, and toll-like receptor 9 (TLR9) may provide potential genes related to resistance against KHV (another term for CyHV-3) (9). However, the magnitude of type I IFN response did not correlate with a higher resistance in CyHV-3-infected carp, during the challenge test among different strains, although CyHV-3 infection can induce type I IFNs (7, 10). Regarding the innate resistance in carp, the mapped CyHV-3 survival quantitative trait loci have been reported mainly in *IL10* and *MHC II* (11). Recently, by quantitative trait locus mapping and genome-wide association study, *tumor necrosis factor-alpha* (*tnfa*), *hypoxia inducible factor 1 subunit alpha* (*hif1a*), *galectin-8* (*LGALS8*), *rootletin*, and *paladin*, have also been related to resistance against CyHV-3 (12). Adaptive immunity through both cytotoxicity and immunoglobulin (Ig) secretion may be involved in resistance. Matthew et al. revealed that, in the anterior kidney, Ig secretion plays an important role in the resistance during the persistent infection or reactivation phases of CyHV-3 (1). In addition, CyHV-3 profoundly influences the expression of host miRNA, although the regulation of immune processes by miRNA in the clinical and latent phases differs (4). This suggests an important role of non-coding RNAs in anti-CyHV-3 immunity.

Transcriptomic studies have been a widely used tool to reveal molecular pathology (13, 14) and even pathogen discovery (15) during fish viral infection. This could facilitate understanding the pathogenic mechanisms of diseases and the immune system of fish (16). For CyHV-3 infection, the first transcriptional analysis of carp anterior kidney mainly pointed out the important role of humoral immune responses, especially those related to immunoglobulin (1). Spleen transcriptomic analyses comparing the susceptible and resistant common carp revealed that the susceptible fish elicited a typical anti-viral interferon response, while the upregulated IL-8 attracted innate immune cells and related response may play an essential role in resistant fish (17). Recent studies have demonstrated that lncRNAs are widely modulated during fish viral or bacterial infections (18–21). As for virus infection, lncRNAs could be involved in regulating the host response during ISAV infection in salmon (18). In zebrafish, SVCV could induce both immune and antiviral processes related to LncRNA (21). The widespread differential expression of lncRNAs in response to infections with different types of pathogens suggested that lncRNAs are pivotal players during immune responses in fish. However, the specific LncRNA modulation of the immune response during CyHV-3 infection has not been accessed in carp.

German mirror carp selection G_4_ is a strain of common carp cultivated widely in China, yet with high mortality caused by the CyHV-3 virus. Based on our previous study, a strain of common carp from German mirror carp selection G_4_ has already shown a higher survival rate after breeding for three generations. Among fish from the G_3_ generation, 1,000 individuals were genotyped by four SNP loci, including carp065309, carp070076, carp183811 and carp160380, and then four main groups, with genotypes of GG/GT/GG/AA, GG/TT/TT/AA, AG/TT/AG/AT and GG/GG/GG/AA, whose survival rates were 89.9, 94.7, 88.0 and 92.7% respectively, were propagated (22). While the unselected mirror carp was 62.6%. Since decreased viral load in tissues directly indicates resistance to CyHV-3 (23), the fact that it has displayed a better immunological index as well as a reduced virus load in immune organs, such as the kidneys and spleen (24, 25), strongly suggests resistance to CyHV-3 in current breeding strain. In detail, acid phosphatase in the spleen, glutathione and total antioxidation capacity in the kidney, lysozyme and immunoglobulin M in the serum, and alkaline phosphatase in the spleen and kidney also showed significant differences between G_1_ and G_3_ or G_1_ and G_2_/G_3_. Meanwhile, the survival rate after the CyHV-3 challenge increased generation by generation. The strong resistance to CyHV-3 has been stable for G_3_ (24). Recently, Sun has revealed that virus genes *TK* and *ORF72* in the G_4_ were expressed at levels significantly lower than those in the non-breeding strain (25). Thus, the resistance to CyHV-3 in G_4_ was strongly deduced.

However, there is a lack of systemic studies focusing on a detailed network of the immune system for the anti-CyHV-3 immune mechanism in this resistant strain. The third-generation sequencing method, such as PacBio, can decode the genetic sequences that are markedly longer compared with the second-generation method, such as Illumina (26). Therefore, a higher quality assembly of transcripts, including both mRNA and non-coding RNAs, may provide a more detailed understanding of the mechanism of genetic resistance.

**Figure 1:**
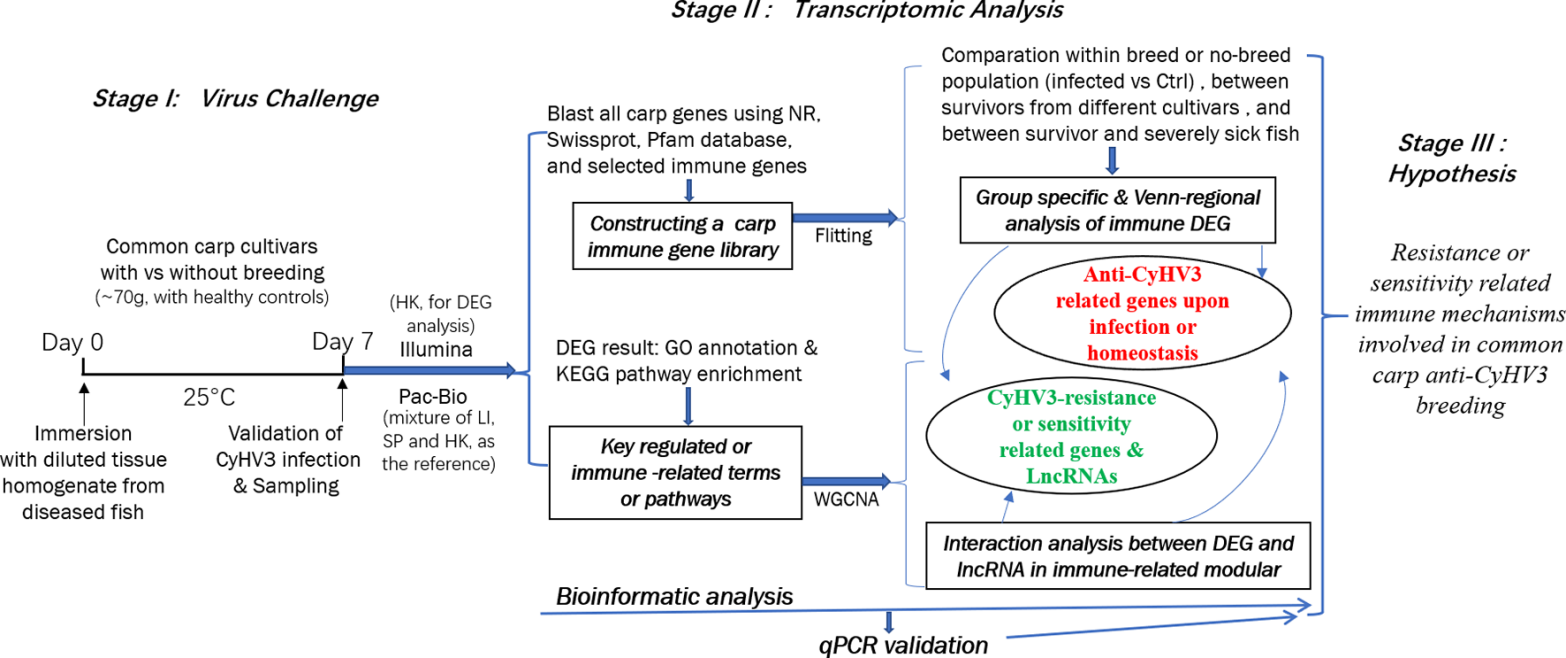
The strategy of integrative transcriptomic analysis for revealing immune mechanisms involved for resistance or sensitivity for current anti-CyHV3 breeding in common carp. During stage I, the disease modelling for CyHV3 infection was established using immersion of virus-containing tissue homogenate and then validated by checking the expression of virus genes. The transcriptomic sampling was done on day 7 post-infection for sequencing using both Illumina and Pac-Bio platforms. In stage II, the bioinformatic analysis was carried out for the transcriptomic data. DEG was generated from five comparison groups, including SvC-B, SvC-N, SvS-BvN-D7, CvC-BvN-D0, and SvI-B&N. The fish with severe illness swam very slowly or even floated on the water. The revealed DEGs were annotated by GO terms and KEGG pathways. Further, a common carp immune gene library was constructed for further immune-related analysis. Additionally, to reveal the LncRNA regulation, WGCNA was used to reveal the interaction between differential expressed transcripts and LncRNA in immune-related module. Thereafter, the resistance or sensitivity related immune mechanisms involved in common carp anti-CyHV3 breeding could be enlightened accordingly.

In this study, to reveal the immune mechanisms involved in anti-CyHV3 breeding for common carp, integrative transcriptomic analysis was performed for both mRNA and long non-coding RNA (lncRNA) during the CyHV-3 challenge, using the strategy shown in **Figure 1**. Immune-related transcripts, from both survivors and healthy controls of either breeding or non-breeding strains, were analyzed. By comparing data from different groups for different strains obtained through both Illumina (New England Biolabs, Ipswich, MA, USA) and Pacific Biosciences (PacBio) sequencing, both immune-related differentially expressed genes (DEG) and lncRNA have been revealed for the Kyoto Encyclopedia of Genes and Genomes (KEGG) pathways and/or Gene Ontology (GO) terms involved. The immune gene-related lncRNA was also explored. This study may shed some light on molecular breeding for virus-resistant fish strains.

## Materials and Methods

### Ethics Statement

All procedures involving animals in this study were conducted according to the guidelines for the care and use of laboratory animals of Heilongjiang River Fisheries Research Institute, Chinese Academy of Fishery Sciences (Harbin, China). The studies involving animals were reviewed and approved by the Committee for the Welfare and Ethics of Laboratory Animals of Heilongjiang River Fisheries Research Institute, Chinese Academy of Fishery Sciences.

### Animals and Virus Challenge

German mirror carp selection G_4_ used in this study were obtained from Kuandian Research Base of Heilongjiang River Fisheries Research Institute (Liaoning, China). The breeding strain was the fourth generation after the selection of resistance to CyHV-3 (25). Two G_4_ groups with survival rates of 94.7 and 92.7% were mixed with the ratio 1:1 and used as the experimental populations in this study. Both the breeding strain G_4_ and non-breeding strain were used for the virus challenge. Since CyHV-3 has a mucosal route of infection mainly *via* the skin rather than the gut (2), this study induced infection with CyHV-3 by adding the homogenate solution of internal organs from sick fish into the water of the tanks following the previously published methods (24, 25, 27). The homogenate solution was prepared using organs from 10 severely sick fish, which swam very slowly or floated on the water. Before using the homogenate to infect fish, the head kidney tissues from those sick fish were checked for the CyHV-3 infection by PCR of virus genes *TK* and *Sph* following the method described in the industry-standard SC/T 7212.1-2011 (28). The CyHV-3 that contained internal organs from sick fish was homogenized to generate 100 ml homogenate, which contained 1.5 × 10^7^ copy CyHV-3 per mg homogenate by PCR-checking virus gene *Sph.* Then the homogenate was used to infect the experimental fish, 17 ml homogenate was used for each tank. For either the breeding or non-breeding strains, the virus challenging group (using diseased fish tissue’s homogenate) was paralleled with the control group (using saline instead). For each treatment groups, three replicates have been done. In each tank (1.6 m × 1.2 m × 0.6 m), which contained around 1 m^3^ water, healthy juvenile common carp (~70 g; N = 100 fish) were used. The water temperature during the experiment was maintained at 25 ± 1°C.

### Sampling and Pathological Analysis

A selection of common carp, from either the breeding or non-breeding strains, were sacrificed. Head kidney samples were obtained at days 0 and 7 after challenge, representing the control and survivor carp. For each tissue sample, three fish were used. For transcriptomic analysis, the samples (N = 3) were collected immediately and soaked in 10 volumes of RNAlater (Qiagen, Hilden, Germany), for sequencing using Illumina (New England Biolabs). Further, to better sequence and generate the lncRNAs, PacBio sequencing was applied to analyze the mixture of liver, spleen, and kidney, with two sample replicates. Additionally, for the validation of transcriptomic data, the qPCR samples (N = 3) were also collected using RNAlater. The number of dead fish during the experiment was counted daily to calculate the mortality rate. Meanwhile, to analyze the degree of swelling of inner organs, the proportion of trunk kidney to the whole trunk area was calculated by ImageJ (https://imagej.en.softonic.com/). In detail, the area of both trunk kidney and trunk regions were selected and measured, and the percentages between them were calculated based on eight survivors of breeding or non-breeding strain. T-tests were applied to test the significance of the difference.

### RNA Extraction

RNA was isolated using the AllPrep DNA/RNA FFPE Kit (Qiagen, Hilden, Germany), according to the instructions provided by the manufacturer. RNA degradation and contamination were monitored on 1% agarose gels. RNA purity was checked using the NanoPhotometer spectrophotometer (Implen Inc., Westlake Village, CA, USA). RNA concentration was measured using the Qubit RNA Assay Kit in a Qubit 2.0 Flurometer (Life Technologies, Carlsbad, CA, USA). RNA integrity was assessed using the RNA Nano 6000 Assay Kit of the Agilent Bioanalyzer 2100 system (Agilent Technologies, Santa Clara, CA, USA).

### Library Preparation and Sequencing for Transcriptomic Analysis

Optimized RNA-Seq strategies, including both PacBio and Illumina (New England Biolabs) sequencing (29), were used to more precisely resolve the sequence of transcripts. Firstly, the total RNA isolated from the head kidney was used to construct the cDNA library. Subsequently, the library was sequenced on a PacBio RS II platform (Biomarker Technologies, Beijing, China). For the PacBio Long Read Processing, raw reads were processed into error-corrected reads of insert using Iso-seq pipeline with minFullPass = 0 and minPredictedAccuracy = 0.90. Next, full-length, non-chemiric transcripts were determined by searching for the polyA tail signal and the 5’ and 3’ cDNA primers in reads of the insert. Iterative Clustering for Error Correction (ICE) was used to obtain consensus isoforms, and full-length consensus sequences from ICE were polished using Quiver. High-quality transcripts with post-correction accuracy of >99% were retained for further analysis.

The illustration of transcripts obtained from the results of PacBio could provide reference transcriptional sequences for the assembly of Illumine sequencing data, to improve sequencing quality. Therefore, Illumina (New England Biolabs) sequencing was performed on all head kidney samples. The procedure used for the preparation of the gene library and sequencing of the transcriptome followed previously published methods (30). Briefly, sequencing libraries were generated using the NEBNext UltraTM RNA Library Prep Kit for Illumina (New England Biolabs), and the library quality was assessed on the Agilent Bioanalyzer 2100 system. The library preparations were sequenced on an Illumina platform, and 150 bp paired-end reads were generated.

### Annotation and Functional Analysis of Transcripts by Public Databases

All reads in the transcriptome data were mapped on the common carp genome (https://asia.ensembl.org/Cyprinus_carpio_german_mirror/Info/Index) (31) for annotation. In detail, GMAP (Genomic Mapping and Alignment Program) and BLAST (version 2.2.26) were applied for mapping the transcriptome data to carp genome and obtaining annotation respectively. The data from the Illumina platform were also used to check and replace errors by proovread (version 2.14.1) in the data from the PacBio platform. Both the annotation of genes and lncRNAs were subsequently generated. The protein-coding transcripts were annotated by NR, swissprot and Pfam database. DEGs were detected from five comparison groups, including intra-strain comparison groups SvC-B (survivors *vs.* controls in the breeding strain) and SvC-N (survivors *vs.* controls in the non-breeding strain), inter-strain comparison groups SvS-BvN-D7 (the comparison between survivors at day 7 for the two strains to compare survivors from the breeding to non-breeding strain) and CvC-BvN-D0 (the comparison between control fish at day 0 for the two strains to compare controls from the breeding to non-breeding strain), and group SvI-B&N. The vital genes for survival were investigated through group SvI-B&N, in which the DEGs with no significant differences (*p >*0.05) were compared between the survivors from the two strains with the transcripts from fish with severe illness. The revealed DEGs were annotated by GO terms and KEGG pathways, following a previously published protocol (30, 32). In brief, functional annotation and the classification of genes were determined by both employing local genes blasts against GO Consortium (http://geneontology.org/) and KEGG (https://www.kegg.jp/kegg/pathway.html). Enrichment of the KEGG pathways was carried out for both upregulated and downregulated genes for all comparison groups. Then to demonstrate the immune DEGs involved pathways more clearly, the gene list of the current construct common carp immune gene library was used as follows. In addition, the LncRNAs were also annotated by GO terms.

### Construction of the Common Carp Immune Gene Library

The common carp immune library was constructed by following our previously published method, which was applied to grass carp (30) and tilapia (32), with some adjustment for viral infection-related immune genes. The modifications were based on gene information obtained by blasting each sequence to databases, including NCBI NR database, as well as Swiss-Prot and Pfam databases. The common carp immune gene library contained information for immune genes at two levels. For the first level, nine categories of immune processes, namely “acute phase reactions”, “pattern recognition”, “antigen processing and regulators”, “complement system”, “inflammatory cytokines and receptors”, “adapters, effectors and signal transducers”, “innate immune cells related”, “T/B cell antigen activation”, and “other genes related to immune response”, were proposed. Subsequently, many categories of immune genes for each immune process (detailed in **Table S1**) were applied for the second level. The library was used to filter transcriptome data and obtain details of the immune processes and particular immune genes for each comparison group, during the GO term and KEGG pathway enrichment.

### Statistical Analysis

The DEG was generated by comparing the RPKM (Reads Per Kilobase of transcript, per Million mapped reads) using the DESeq2 R package (1.16.1). The resulting *p* values were adjusted using the Benjamini and Hochberg approach for controlling the false discovery rate. Genes with an adjusted *p <*0.05 found by DESeq2 were assigned as being differentially expressed. The following bioinformatics analysis was performed to select immune-related transcripts from the common carp immune gene library and construct the bar plots for the major immune processes and immune categories. The t-test was used to assess differences, with a false discovery rate adjusted *p <*0.05. Qualitative comparisons were performed between samples by counting the number of DEG. The data were rearranged in Microsoft EXCEL, and applied to plot charts by ggplot2 (2.2.1) using R language.

### Correlation Analysis Between LncRNA and Genes Involved in Immune-Related GO Terms

The weighted gene co-expression network analysis (WGCNA) was performed using the R package “WGCNA” (33). Specifically, all genes with an expression variance ranked in the top 75 percentile of the data set were retained (34). The R package WGCNA was used to construct the weighted gene co-expression network (35). A matrix of signed Pearson correlations between all gene pairs was computed, and the transformed matrix was used as input for linkage hierarchical clustering. All transcripts in current transcriptome data with similar expression patterns were clustered together as one module. Subsequently, using the R package clusterProfiler (36), enriched GO terms for lncRNA-related protein-coding genes were generated for the DET list of every module. The *p* values of enriched GO terms were produced from the Kolmogorov–Smirnov test. To elucidate the detailed lncRNA-mRNA network, the immune-related GO term containing module was selected, and the relationship of involved top 100 transcripts and related lncRNAs were shown by the cystoscope software. In addition, the immune DETs (differentially expressed transcripts) involved in the immune-related module were classified into comparison groups SvC-B, SvC-N and SvS-BvN-D7, to reveal the LncRNA modulation of immunity at the aspect of surviving from CyHV-3 infection.

### Quantitative Reverse Transcription Polymerase Chain Reaction (qPCR)

The mRNA samples used for transcriptome sequencing were also subjected to qPCR validation (n = 3), using SYBR Green PCR master mix (Bio-Rad) and CFX real-time PCR detection system (Bio-Rad), following a previously published protocol (37, 38). After RNA isolation, reverse transcription and qPCR experiments were carried out for 11 genes. Gene-specific primers (**Table S4**) were designed with the Primer Premier 5.0 software. Housekeeping gene 18s RNA was used to normalize data, and 2-DDCt formula was used to calculate the relative expression.

## Results

### Virus Identification, Mortality Rate and Pathological Appearance

**Figure 2:**
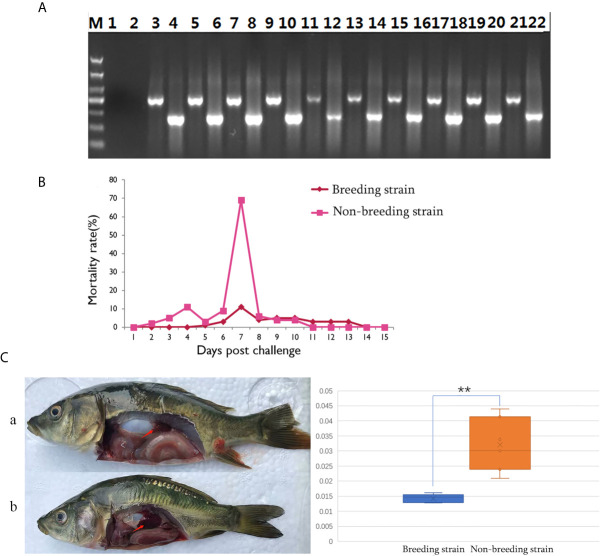
Differential appearance of general mortality and pathology between fish from the breeding and non-breeding strains. **(A)** The PCR validation of the CyHV-3 virus genes TK and Sph. Lane 1-2 represents the negative control, and afterwards, lane 3-22 represents the result for tested 10 virus infected fish. The lanes of odd and even numbers showed the PCR result of TK and Sph genes, respectively. M is the abbreviation of “marker”. **(B)** Comparison of daily mortality between the breeding and non-breeding strains. The mortality was monitored within 15 days post challenge. N=300 per group. **(C)** The degree of swelling trunk kidney was limited in the survivors from the breeding strain (a) compared with the markedly enlarged trunk kidney observed in fish from the non-breeding strain (b). The arrow indicates the trunk kidney region. “**” means the very significant difference (p < 0.01) between current compared two groups.

The successful infection of CyHV-3 has been validated using PCR, to check the expression of virus genes (*TK* and *Sph*), in randomly sampled severely sick fish (N = 10) at day 7 after challenge (**Figure 2A**). Further, to reveal the mortality, the number of dead fish was recorded from days 1 to 15 after challenge (**Table S1**), the mortality rate was then calculated for all groups (**Table S2**). For the challenged non-breeding strain (**Figure 1B**), the mortality rate increased daily, except for a decrease observed on day 5. Mortality peaked at day 7 (69%) and decreased to a markedly low level thereafter. For the challenged breeding strain (**Figure 2B**), the mortality rate remained at a markable low level, with the highest value (11%) recorded at day 7. The mortality rates of the two challenged groups were very significantly different (*p <*0.01). The number of dead fish for each day is listed in **Table 1** for the breeding or non-breeding strain, while in the two unchallenged controls, there were no dead fish (data not shown). Compared with the organs in survivors of the breeding strain, pathological swelling and hyperemia of the internal organs (particularly the kidney) were obvious in survivors from the non-breeding strain on day 7 (**Figure 2C**). In detail, the proportion of kidney area to the whole trunk area in the breeding strain was significantly different from that of the non-breeding strain (*p <*0.01) (**Figure 2D**). The reduced immune organ swelling was reflected by the ratio of 0.44 comparing the proportions between the breeding and non-breeding strains (**Table S2**).

**Table 1:** The number of dead fish for each day during the CyHV-3 challenging for breeding or non-breeding stain.

Stain	D1	D2	D3	D4	D5	D6	D7	D8	D9	D10	D11	D12	D13	D14	D15
Breeding	0	0	0	0	1	3	11	4	5	5	3	3	3	0	0
Non-breeding	0	2	5	11	3	9	69	6	4	4	0	0	0	0	0

### Quality and Validation of Transcriptomic Data

Sequencing was performed with both the PacBio RS II and Illumina platforms to analyze the gene information of the common carp. Through PacBio sequencing, 19.49 G data were obtained. Of the 258,346 ccs, 79.79% were full-length sequences, and 17,769 polished high-quality isoforms were also revealed (**Table S3**, sheet 1). Meanwhile, the Illumina data were also characterized by high quality, and the information for each sample is provided in **Table S3** (sheet 2). To validate the transcriptomic data, qPCR was conducted. A total of 24 reactions were performed to validate the transcripts of 11 genes, most of which were immune genes. The correlation analysis showed that fold changes between transcriptome and qPCR results correlated well (R^2^ = 0.929718564). The gene ID, annotation, primers, and fold-change information are listed in **Table S4**.

### GO Analysis of Immune-Related DEGs Among Tested Fish

**Figure 3:**
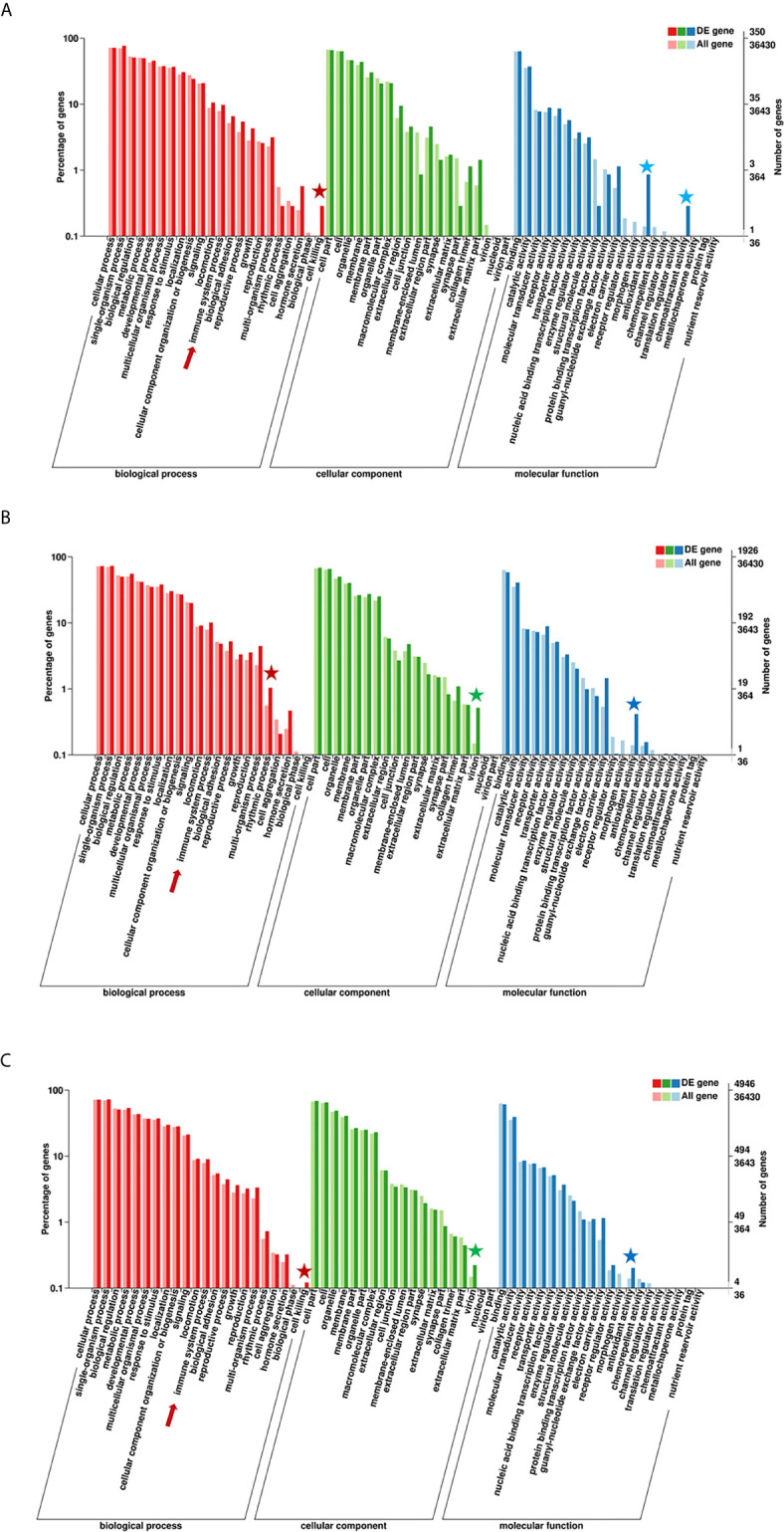
GO (Gene Ontology) terms of comparison groups SvC-B, SvC-N, and SvS-BvN-D7. The different colors of the bar indicate the comparison of the GO terms between all genes and immune-related genes. In detail, red and pink bars represent all genes and immune-related genes respectively for the biological process (BP, in part **A**), green and light green bars represent those for the cellular component (CC, in part **B**), blue and light blue bars represent those for the molecular function (MF, in part **C**). The immune-related terms are labeled with stars.

According to the enriched general GO terms (**Figure 3**) of immune-related DEGs, there are “immune system process” and “antioxidant activity” for all comparison groups, “chemoattractant activity” only in comparison group SvC-B, “rhythmic process” only in comparison group SvC-N, “cell killing” in both comparison groups SvC-B and SvS-BvN-D7, and “virion” in both comparison groups SvC-N and SvS-BvN-D7. The detailed significant enriched GO terms involved in biological processes (BP), cellular components (CC), and molecular functions (MF) were detailed in **Tables S5–S9** for all comparison groups. The immune-related significant regulated BP terms (*p <*0.01) were in the largest number (**Table 2**), for example, “positive regulation of cell migration” in group SvC-B; “activation of MAPK activity”, “regulation of protein ubiquitination involved in ubiquitin-dependent protein catabolic process”, “oxication-reduction process” and “positive regulation of cell migration” in group SvC-N; “activation of MAPK activity” and “dopaminergic neuron differentiation” in group SvS-BvN-D7. Besides, the CC terms (*p <*0.01) includes “Pre-autophagosomal structure membrane” in group SvC-B, SvC-N, CvC-BvN-D0 and SvI-B&N, and the MF terms (*p <*0.01) includes “NF-KappaB binding” and “lysozyme activity” in group SvC-N; “cytokine activity” in group SvS-BvN-D7; “Lysozyme activity”, “MAP kinase activity” and “ubiquitin-like protein binding” in group CvC-BvN-D0.

**Table 2:** Featured immune-related GO biological process terms for all comparison groups.

Compared group	GO terms (*P < *0.01).
Group SvC-B	Phagocytosis, positive regulation of immune system process, positive regulation of I-kappa B kunase/NF-kappaB signaling, integrin-mediated signaling pathway, regulation of B cell activation, macrophage activation, lymphocyte-mediated immunity
Group SvC-N	Response to lipopolysaccharide, regulation of granulocyte differentiation, toll-like receptor signaling pathway, positive regulation of immune system process, macrophage differentiation, phagocytosis, engulfment, lymphocyte activation, integrin-mediated signaling pathway, leukocyte activation
Group SvS-BvN-D7	Response to lipopolysaccharide, negative regulation of B cell apoptotic process, positive regulation of TOR signaling, macrophage differentiation, endosome to lysosome transport, regulation of granulocyte differentiation, toll-like receptor signaling pathway, defense response to fungus, phagocytosis, engulfment, T cell proliferation, mast cell activation, myeloid cell activation involved in immune response, regulation of T cell differentiation in thymus, phagosome maturation, positive regulation of T cell activation
Group CvC-BvN-D0	Phagocytosis, positive regulation of immune system process, positive regulation of I-kappaB kinase/NF-kappaB signaling, integrin mediated signaling pathway, regulation of B cell activation, lymphocyte mediated immunity, macrophage activation, response to lipopolysaccharide
Group SvI-B&N	Phagocytosis, positive regulation of cell migration, positive regulation of I-kappaB kinase/NF-kappaB signaling, positive regulation of myeloid leukocyte differentiation, integrin-mediated signaling pathway, regulation of B cell activation, leukocyte activation, regulation of immune response, and macrophage activation

The details of the GO terms involved in biological process were listed in **Tables S5**–**S9**.

### KEGG Analysis of DEGs Among Tested Fish

**Figure 4:**
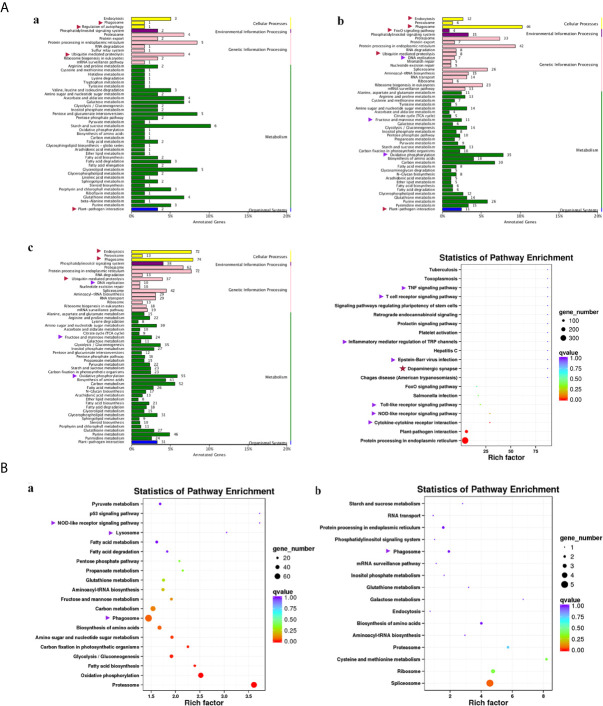
KEGG (Kyoto Encyclopedia of Genes and Genomes) pathways revealed for all comparison groups. In part **(A)**, the enriched pathways for comparison group SvC-B (a), SvC-N (b) and SvS-BvN-D7 (c) were shown mainly in bar plots. In addition, for the comparison group SvS-BvN-D7, the babble plot of pathways for immune-related DEG was also given in the right to illustrate the rich factor. In part **(B)**, the pathways of immune-related DEG for both comparison group CvC-BvN-D0 (a), which compared controls of both stains, and group SvI-B&N (b), which has compared similar genes of survivors from both strains with protein-coding transcripts of severely sick fish, were demonstrated in babble plots. The immune-related terms are labeled with arrows. Red and purple arrows indicate the immune and related pathways representatively.

Compared with group SvC-B, there were markedly more genes involved in immune and related pathways in group SvC-N. Current revealed immune pathways in group SvC-B were “phagosome”, “regulation of autophagy”, “ubiquitin mediated proteolysis”, and “plant-pathogen interaction” (**Figure 4Aa**). Meanwhile, there are four immune pathways, including “endocytosis”, “phagosome”, “FoxO signaling pathway”, “ubiquitin mediated proteolysis”, “DNA replication”, “fructose and mannose metabolism”, “oxidative phosphorylation”, and “plant–pathogen interaction” in group SvC-N (**Figure 4Ab**). To get a better understanding of the different survival mechanism, the pathways revealed in group SvS-BvN-D7 includes “endocytosis”, “phagosome”, “ubiquitin mediated proteolysis”, “DNA replication”, “fructose and mannose metabolism”, and “oxidative phosphorylation” (**Figure 4Ac**, left).

To better illustrate the immune and related pathways more clearly, filtering of DEG was conducted using the gene list of the common carp immune gene library (**Table S1**), and then a bubble chart was used to clarify both the enrichment factors and gene numbers for revealed immune pathways. For group SvS-BvN-D7, which reflected differential surviving mechanism [**Figure 4A** (c, right)], the revealed immune pathways include “TNF signaling pathway”, “T cell receptor signaling pathway”, “inflammatory mediator regulation of TRP channels”, “Epstein–Barr virus infection”, “Toll-like receptor signaling pathway”, “NOD-like receptor signaling pathway” and “cytokine-cytokine receptor interaction”. In addition, “dopaminergic synapse” was also found. While, groups CvC-BvN-D0 and SvI-B&N, revealed the difference and similarity for maintaining basic homeostasis, respectively. The most enriched immune pathways were the “p53 signaling pathway”, “NOD-like receptor signaling pathway”, “lysosome”, “oxidative phosphorylation”, and “proteasome” in group CvC-BvN-D0 (**Figure 4Ba**), yet only “phagosome” in group SvI-B&N (**Figure 4Bb**).

### Classification of DEG From Different Comparison Groups Into Immune Process and Immune Gene Category

**Figure 5:**
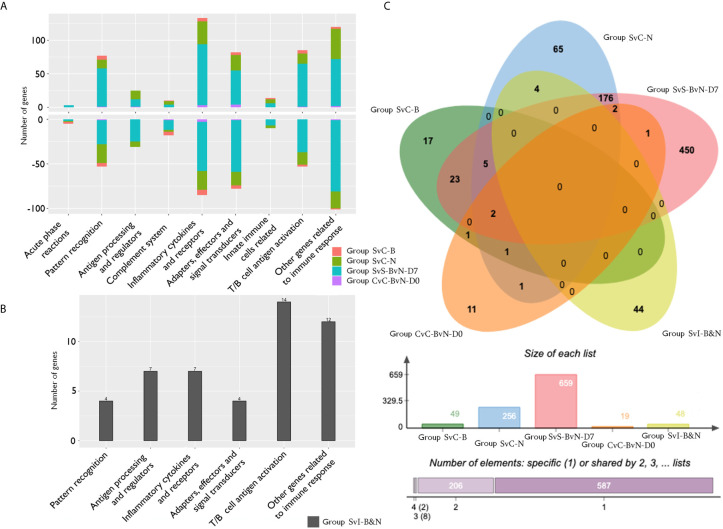
The analysis of immune processes of DEG among different comparison groups. **(A)** The bar plots of gene number for the involved immune processes of up or down-regulated DEGs in groups SvC-B, SvC-N, SvS-BvN-D7, and CvC-BvN-D0; **(B)** The barplot of gene number for the involved immune processes in group SvI-B&N; **(C)** The Venn diagram of gene number for all comparison groups. DEG, differentially expressed gene.

The current construction of the common carp immune gene library (**Table S4**) was used to classify the revealed immune transcripts and refine the involved immune processes and immune gene categories. The details of DEG involved in all comparison groups were provided in **Tables S5–S9**. At the level of immune processing, in group SvC-B (**Figure 5A**, **Tables 3A** and **S5**), most immune mRNAs were upregulated in the immune processes of “pattern recognition”, “inflammatory cytokines and receptors”, and “T/B cell antigen activation”, while downregulated in the immune process of “inflammatory cytokines and receptors”, “complement system”, “adapters, effectors and signal transducers”, and “pattern recognition”. Interestingly, there were no genes in “antigen processing and regulators” for group SvC-B. In addition, there were only downregulated genes in “acute phase reactions”, as well as only upregulated genes in “innate immune cells related” in group SvC-B. In group SvC-N (**Figure 5A**, **Tables 3A** and **S6**), immune genes were upregulated in the immune processes, such as “other genes related to immune response”, “inflammatory cytokines and receptors”, and “adapters, effectors and signal transducers”. Meanwhile, the downregulated genes were involved in the immune processes, such as “pattern recognition”, “inflammatory cytokines and receptors” and “other genes related immune response”. The largest number of immune mRNAs was observed in group SvS-BvN-D7 (**Figure 5A**, **Tables 3A**, and **S7**). The upregulated immune DEGs (BS-advantage) were mainly involved in “inflammatory cytokines and receptors”, “other genes related to immune response”, “T/B cell antigen activation”, “pattern recognition”, and “adapters, effectors and signal transducers”, whereas the downregulated immune DEGs (NBS-advantage) were mainly involved in “other genes related to immune response”, “adapters, effectors and signal transducers”, “inflammatory cytokines and receptors”, “T/B cell antigen activation”, “pattern recognition”, and “antigen processing and regulators”. Meanwhile, in group CvC-BvN-D0 (**Figure 5A**, **Tables 3B** and **S8**), the upregulated immune DEGs (BS-advantage), were found mostly in “adapters, effectors and signal transducers”, and noted in “antigen processing and regulators” and “T/B cell antigen activation”, while downregulated immune DEGs (NBS-advantage), were mostly involved in “inflammatory cytokines and receptors”, and also noted in “pattern recognition” and “complement system”. In group SvI-B&N (**Figure 5B**, **Tables 3B** and **S9**), among the revealed six processes, “T/B cell antigen activation” and “other genes related to immune response” exhibited the greatest DEG number. To illustrate the immune process, the involved immune gene categories of the immune-related DEG in all comparisons were listed in **Table 3**.

**Table 3A:** Immune gene categories involved in differentially expressed mRNA for comparison groups SvC-B, SvC-N, and SvS-BvN-D7.

Compared group	Immune gene category	
	1<FC<2	2<FC
Up-regulated in group SvC-B (26)	LRR-containing proteins, MRC, NFIL3, perforin, UBE, PIGR, TRIM, TLX.	CSF, UBE, C3, GVIN, IL21R, IL23R, integrin alpha, MMR, NCAM, cathepsin, ubiquitin ligase ladderlectin, LRR-containing proteins, Ig heavy chain, Ig light chain, PIGR, SEMA.
Down-regulated in group SvC-B (23)	CXCL, integrin alpha, myeloid cell related, galectin.	Fibrinogen, NALP12, NLRC3, C3, GVIN, IFI, IL1R, C-type lectin, galectin, TLR18, Ig light chain, TIP.
Up-regulated in group SvC-N (155)	FBXL, fucolectin, MPR, nattectin, TLR1, TLR5, CDK, CDK related, CDKIs, HA, MHC I, MHC II, TGFB, TNF, TNFAIP3-interacting protein, C3aR, C7, CCL, CCR, CFLAR, CXCL, CXCR, cytokine receptor, IFI, IFIT, IL1R related, IRF8, NFIL3, MAPK, MAPKKK, NALP12, NLRC3, programmed cell death protein, SOCS1, SOCS3, SOCS7, TRAF, TTRAP, UBE, MIF, NCF, NCK and related, BCL, BLNK, FCR, HDAC, HMG, Ig light chain, PBX, TAGAP, AP-3, CARD, cathepsin, CEBP, hepcidin, HSP, lymphocyte antigen, myeloid cell related, PIN1, platelet related, TMED, ubiquitin ligase, ubiquitin related, TRIM, UBL.	MAPK, MAPKKK, NLRC3, MHC II, C3, CCL, CXCL, CXCR, IL-1, IL1R, IL-6, IL6R, IL-8, IRF8, MX, NRAMP, AP-3, cathepsin, HSP5, HSP70, mucin, myeloid cell related, platelet related, ubiquitin ligase, NITR, TLR18, TLR22, TLR25, CD22, HMG, TCIRG1,
Down-regulated in group SvC-N (102)	Plasminogen, C-type lectin, Hepatic lectin, LRR-containing proteins, RBL, SITR, TLR13, CD22, Ig heavy chain, Ig light chain, SEMA, TCR, CDK, VEGF, VEGFR, C3aR, ACRs, CCR, EGFR, FGFR, GVIN, IL17R, IL21R, IL2R, integrin alpha, integrin beta, ACTININ, MALT1, paladin, RTK, TRIM, ubiquitin ligase, UBL, granzyme, MAPKKK, NALP12, NALP3, perforin, UBE, CDK, TRIM.	perforin, CD59, CCL, IL6R, IL-8, IRF related, TITIN, NCAM, CD33, HSP90, Kruppel-like factor, ubiquitin ligase, UBL, collectin, galectin, LRR-containing proteins, B-cell CLL/lymphoma, JAG, NFAT, SEMA
BS-advantage immune DEGs in group SvS-BvN-D7 (357)	Fibrinogen, MBL, collectin, C-type lectin, FBXL, fucolectin, galectin, intelectin, LRR-containing proteins, RBL, TLR8, TLR13, TLR3, TLR9, CDKIs, CIITA, LRMP, TGBR, VEGF, C3, C5, ACRs, CCL, CCR, CXCR, EGF, FGFR, GVIN, IFI, IL10R, IL13R, IL17R, IL1R related, IL21R, IL23R, IL2R, IL6R, IL-8, integrin alpha, integrin beta, IRF4, IRF5, IRF7, MX, XCR, CBX4, CSFR, granzyme, MAPK, MAPKKK, NALP12, NALP3, NDRG1, perforin, Programmed cell death protein, TRAF, TRAIL, UBE, MMR, NCAM, B-cell CLL/lymphoma, CD2, CD22, CD276, CD6, FCR, GATA and related, Ig heavy chain, Ig light chain, immunoglobulin superfamily, JAG, LAG, MAL, NFAT, PIGR, PKC, SEMA, T-bet, VTCN, ZAP, ACTININ, cathepsin, CD302, lymphocyte antigen, lymphocyte related, myeloid cell related, nectin, palladin, PI3K, TIAM, ubiquitin ligase, ubiquitin related, TRIM.	Fibrinogen, CSF, granzyme, MAPKKK, NALP12, NALP3, NLRC3, EGF, perforin, TRAF, UBE, CDK, LRMP, MHC I, MHC II, C3aR, C8, CCL, CCR, FGFR, GVIN, IFI, IFIT, IL17R, IL1R related, IL21R, IL23R, IL2R, IL6R, IL-8, integrin alpha, integrin beta, NFIL3, TITIN, MCP, NCAM, cathepsin, HSPb1, lymphocyte antigen, mucin, PI3K, rootletin, RTK, TRIM, ubiquitin ligase, ubiquitin related, UBL, collectin, c-type lectin, fucolectin, Lgals3bpa, galectin, Hepatic lectin, ladderlectin, LRR-containing proteins, MRC, NITR, scavenger receptor, SIGLEC, CD22, CD4, FCR, HDAC, Ig heavy chain, Ig light chain, immunoglobulin superfamily, JAG, NFAT, PIGR, PKC, SEMA, VSIG.
NBS-advantage immune DEGs in group SvS-BvN-D7 (303)	c-type lectin, FBXL, LPS-anchor protein, LRR-containing proteins, MPR, TLR1, TLR22, CDK, CDK related, CDKIs, HA, MHC II, TGFB, TNFAIP3-interacting protein, C3, C3aR, C4, C7, ACRs, CD11, CFLAR, CXCR, cytokine receptor, FAM, GVIN, HB-EGF, IL12R, IL1R related, IL-6, IL6R, integrin alpha, integrin beta, IRF1, IRF8, NFIL3, CSFR, JAK1, MAPK, MAPKKK, NALP12, NALP3, NLRC3, Programmed cell death protein, SOCS1, SOCS3, SOCS7, STAT3, UBE, MIF, NCF, NCK and related, BAFF, B-cell CLL/lymphoma, BCL, BLNK, CD22, CD276, FCR, HDAC, HMG, Ig light chain, PBX, PIGR, SEMA, TAL, VSIG, WWP, AP-3, CARD, cathepsin, CEBP, HSP, HSP4, HSP5, HSP70, HSP90, Kruppel-like factor, lymphocyte antigen, MALT1, mucin, myeloid cell related, PAXILLIN, PIN1, platelet related, TMED, ubiquitin ligase, ubiquitin related, TRIM, UBL.	Macroglobulin, CSF, MAPK, MAPKKK, NALP12, NALP3, NLRC3, SOCS3, UBE, CDK, CDKIs, MHC II, TNF, FGF, TNFAIP3-interacting protein, C3, C3aR, C7, CD59, CCL, CCR, CXCL, CXCR, GVIN, IL-1, IL-12, IL1R, IL1R related, IL2R, IL-6, IL-8, integrin alpha, IRF8, NFIL3, NRAMP, ACTININ, AP-3, caspase, cathepsin, CEBP, hepcidin, HSP70, lymphocyte antigen, ubiquitin ligase, UBL, CD209, C-type lectin, galectin, intelectin, LRR-containing proteins, TLR18, TLR25, TLR4, TLR5, BCL, CD22, CD276, HDAC, Ig light chain, PIGR, SEMA, TAGAP, TCIRG1, TCIRG2, TCIRG3.

For group SvS-BvN-D7, “BS-advantage” represented the upregulated genes related to the breeding strain, while “NBS-advantage” represented the downregulated genes related to non-breeding strain, after the comparison between survivors from breeding strain and survivors from the non-breeding strain.

**Table 3B:** Immune gene categories involved in differentially expressed mRNA each comparison group CvC-BvN-D0 and SvI-B&N.

Compared group	Immune gene category	
BS-advantage immune DEGs in group CvC-BvN-D0 (12)	1<FC<2: MHC I, GVIN, IL21R, NLRC3;	
	2<FC: NALP3, perforin, STAT3, integrin alpha, platelet related, ubiquitin related, MRC, HDAC.	
NBS-advantage immune DEGs in group CvC-BvN-D0 (7)	1<FC<2: TLR18, C3, CCL, integrin beta.	
	2<FC: perforin, IFI, myeloid cell related	
All in group SvI-B&N (48)	LRR-containing proteins, VEGFR, VEGF, CDKIs, CDK, NFIL3, LIFR, integrin beta, FAM, CCR, CD166, MAPK, CD97, SEMA, PIGR, NFAT, Ig light chain, HMG, HDAC, BTG, BCL, ubiquitin ligase, platelet related, HIF1a, CEBP.	

The number (>1) of DEG is shown in the brackets. The full names of all gene abbreviations could be found in **Tables S5–S9**. The underlined up or down-regulated immune gene categories are the regional specific in the Venn diagram for every group (detailed in **Table S10**). For group CvC-BvN-D0, “BS-advantage” represented the upregulated genes related to the breeding strain, while “NBS-advantage” represented the downregulated genes related to non-breeding strain, after the comparison between controls from breeding strain and controls from the non-breeding strain.

### Venn-Regional Analysis of Immune-Related DEG From Different Comparison Groups

A Venn diagram was created to demonstrate the relationship between the immune-related DEG among all comparison groups (**Figure 5C**). The details for all regions were in **Table S10**. Though the region-specific DEGs accounted for a large proportion of each comparison group, with immune gene categories for region-specific DEG underlined in **Table 3**, the overlapping DEGs (**Table 4**), which contained important genes, were listed with the corresponding immune process and immune gene category.

**Table 4:** The immune process and immune gene category for overlapping genes in the Venn regional analysis.

Overlapping regions	Immune process	Immune gene category
Groups SvC-B & SvS-BvN-D7 (23)	pattern recognition (7), complement system (4), inflammatory cytokines and receptors (3), adapters, effectors and signal transducers (4), innate immune cells related, T/B cell antigen activation (2), other genes related to immune response (2)	CSF, NALP12, UBE, C3, GVIN, IL23R, integrin alpha, NCAM, ubiquitin ligase, C-type lectin, galectin, ladderlectin, LRR-containing proteins, MRC, PIGR
Groups SvC-B & CvC-BvN-D0 (1)	inflammatory cytokines and receptors	IFI
Group SvC-N & SvS-BvN-D7 (176)	pattern recognition (18), antigen processing and regulators (14), complement system (4), inflammatory cytokines and receptors (38), adapters, effectors and signal transducers (23), innate immune cells related (8), T/B cell antigen activation (24), other genes related to immune response (47)	Granzyme, MAPK, MAPKKK, NALP12, NALP3, NLRC3, perforin, Programmed cell death protein, SOCS1, SOCS3, SOCS7, UBE, CDK, CDK related, CDKIs, HA, MHC II, TGFB, TNF, TNFAIP3-interacting protein, VEGF, C3aR, C7, ACRs, CCL, CCR, CFLAR, CXCL, CXCR, cytokine receptor, FGFR, GVIN, IL1, IL17R, IL1R related, IL2R, IL6, IL6R, IL8, integrin alpha, IRF8, NFIL3, TITIN, MIF, NCAM, NCF, NCK and related, NRAMP, ACTININ, AP-3, CARD, cathepsin, CEBP, hepcidin, HSP, HSP5, HSP70,lymphocyte antigen, paladin, PIN1, platelet related, RTK, TMED, TRIM, ubiquitin ligase, ubiquitin related, UBL, collectin, C-type lectin, FBXL, galectin, LRR-containing proteins, MPR, RBL, TLR1, TLR13, TLR22, TLR25, TLR5, BCL, BLNK, CD22, FCR, HDAC, HMG, Ig heavy chain, Ig light chain, JAG, NFAT, PBX, SEMA, TAGAP, TCIRG1
Groups SvC-N & SvI-B&N (4)	antigen processing and regulators (3), other genes related to immune response	CEBP, VEGFR
Groups SvC-N & CvC-BvN-D0 (1)	inflammatory cytokines and receptors	integrin alpha
Groups SvS-BvN-D7 & CvC-BvN-D0 (1)	inflammatory cytokines and receptors	CCL
Groups SvC-B & SvC-N & SvS-BvN-D7 (5)	pattern recognition, inflammatory cytokines and receptors (3), adapters, effectors and signal transducers	LRR-containing proteins, CXCL, IL1R, perforin
Groups SvC-B & SvC-N & SvI-B&N (1)	complement system	C3
Groups SvC-N & SvS-BvN-D7 & CvC-BvN-D0 (2)	adapters, effectors and signal transducers (2)	perforin
Groups SvC-B & SvC-N & SvS-BvN-D7 & CvC-BvN-D0 (2)	pattern recognition, inflammatory cytokines and receptors,	TLR18, IL21R

### Immune-Related GO Terms as Well as Network Correlation Between LncRNAs and Genes in Immune-Related Module

**Figure 6:**
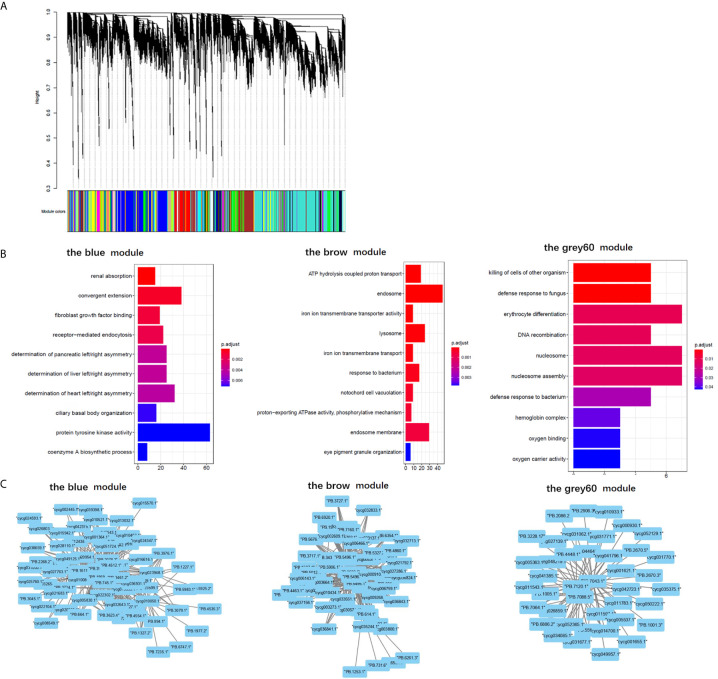
Gene ontology analysis and cystoscopes of key matched lncRNA and transcripts in the immune-related module. **(A)** the cluster dendrogram of all gene module. **(B)** The GO terms involved in the immune-related module, including the blue, brow, and the grey60 module, which contain the matched lncRNA and transcripts involved in current analyzed comparison groups. The star, triangle, and arrow represent the revealed GO term for comparison groups SvC-B, SvC-N, and SvS-BvN-D7, respectively. **(C)** Cystoscopes of key matched lncRNA and transcripts in the immune-related module, as shown for blue, brow and grey60 module, respectively. The PPI network here just showed the pattern of closely related genes and LncRNAs, and to access the detailed information of genes and LncRNAs, please refer to **Table S11**.

The results of “WGCNA” were selected to determine the biological processes involved in CyHV-3-induced modulation of genes by lncRNAs, from an immune perspective. Among the currently divided 35 gene modules (**Figure 6A**), 12 modules were found with immune-related GO terms (**Figure S1**). Generally, “double-stranded DNA binding”, “receptor-mediated endocytosis”, “response to bacterium”, “lysosome”, “defense response to fungus”, “killing of cells of other organism”, “oxygen carrier activity”, “oxygen binding”, “defense response to bacterium”, “killing of cells of other organism”, “NuA4 histone acetyltransferase complex”, “defense response to gram-negative bacterium”, “defense response to gram-positive bacterium”, “immune response”, “regulation of cytokine secretion”, “cytolysis”, “execution phase of apoptosis”, “cellular response to gamma radiation”, “nucleic acid metabolic process”, “TOR signaling”, “DNA replication”, “ubiquitin-dependent protein catabolic process”, “granulocyte differentiation”, and “macrophage differentiation” were revealed after the “WGCNA” analysis. The top five immune-related modules containing most immune-related GO terms (**Figure 6B**) were analyzed for the relationship between LncRNA and transcripts by PPI networks in cystoscope (**Figure 6C**, with details in **Table S11**). The LncRNA regulated genes, which upregulated in group SvS-BvN-D7, such as Semaphorin-4E in the blue module, can be the factor associated with the resistance in the breeding strain. The LncRNA regulated genes, which downregulated in group SvS-BvN-D7, such as Lgals3l in the grey60 module, can be the factor associated with less accessibility to CyHV-3 in the breeding strain. The LncRNA regulated genes, which was upregulated in group SvC-N and downregulated in group SvS-BvN-D7, such as natural resistance-associated macrophage protein (Nramp), plasminogen activator inhibitor (PAI) in the brown module, could be the key clues for susceptibility of CyHV-3 in the non-breeding strain. Specifically, for detailed correlations of lncRNAs and DEG in groups SvC-B, SvC-N, and SvS-BvN-D7 (**Table S12**), T cell leukemia homeobox 3 (TLX3) and LGALS3 were revealed, respectively, in the survivors of the breeding strain.

## Discussion

This study demonstrated that the anti-CyHV-3 immune mechanisms of a breeding strain of common carp. This demonstrated dramatically decreased levels of mortality and less inner tissues swelling together with the previously revealed reduced virus load of tissues (24, 25), proving resistance to CyHV-3. The current studies of biological processes and pathways discovered through transcriptomic analysis have been merged to improve clarity. Afterwards, by comparing the survivors and healthy fish in either breeding or non-breeding strains, key genes and related lncRNAs involved in immune processes were also revealed. Accordingly, the immunogenetically insensitive or susceptible factors to CyHV-3 infection were determined.

From the aspect of cell surface receptors, which could participate in virus-host interaction, current comparison results suggested potential components involved in virus entry and downstream proinflammatory signaling. Firstly, integrin acts as a herpesvirus receptor (39), and the integrin-dependent signalosome in herpesvirus-infected cells mediated or coactivated numerous inflammatory responses and signaling transductions (39). Therefore, the finding that there was more expression of integrin beta 1 (ITGB1) in the survivors from non-breeding strain compared with those from the breeding strain, even during the steady status, may indicate that ITGB1 facilitates the binding of herpes virus glycoprotein for entry (40). This coincides with the finding that integrin signaling promotes the release of intracellular calcium stores and contributes to viral entry and cell-to-cell spreading *via* glycoprotein H, during herpes simplex virus infection in humans (41). Recent reports have also demonstrated that some integrins on lymphocytes, such as B cells, could facilitate mucosa-specific homing (42). Secondly, higher TLR expression in survivors of the non-breeding strain compared with that of the breeding strain also indicated its role in facilitating the virus infection. Of note, TLR4 signaling leads to the production of proinflammatory cytokines in human lymphatic endothelial cells (39). In addition, fish-specific and virus-responding TLR18 (43, 44) (the most overlapping gene among groups) was downregulated in the comparison group SvC-B, whereas it was upregulated in group SvC-N. Also, TLR18 was higher in steady-state in the nonbreeding strain. This suggested its possibility to enhance the severity of CyHV-3 infection. MAPK signaling, as the downstream process of pattern recognition receptors, could facilitate the tnf-alpha-induced suppressor of cytokine signaling 3 (SOCS3) expression. This can lead to both pro-inflammatory immune response and failure in growth (45), according to the upregulated DEGs upon infection observed in the group SvC-N.

To reveal the breeding driven improvement for the resistance from CyHV-3 infection, by comparing the survivors from different strains, DEGs of the inflammatory status also provide clues for how to block the virus. Non-specific binding of the virus by lectins played a protective role in preventing virus entry. Both upregulated ladderlectins and higher galectin expression were detected in survivors of breeding strains compared with non-breeding strains. This is suggestive of their blocking ability for viral proteins (46, 47), such as glycoprotein (48). Moreover, as the head kidney is one of the major reticuloendothelial systems in fish (49), mucin was also found to be upregulated as the anti-virus barrier. This was shown in the comparison group SvC-N as the gel-forming mucin 5B (MUC5B) (50) was upregulated in the survivors of the non-breeding strain. However, higher expression levels of membrane-bound MUC3 (51) were found in survivors of the breeding strain versus the non-breeding strain. Additionally, as shown in the comparison group SvS-BvN-D7, the complement system functioned differently. BS-advantage complement components C8 indicate the formation of terminal complement complex (52), while NBS-advantage CD59, which is the inhibitor of complement membrane attack complex (MAC) (52), suggesting the inhibition of f complement-dependent cytotoxicity (CDC). Thus, the effective CDC may be more helpful for surviving. These findings suggested that more mucus was secreted, thereby causing tissue swelling upon CyHV-3 infection in the non-breeding strain. For the survivors of the breeding strain, the membrane-bound mucin could effectively bind the virus with no gel.

For the anti-viral biological process for the breeding strain, the lectin complement pathway has been involved in the clearance of apoptotic cells, reflected by the GO term “cell killing” in both comparison groups SvC-B and SvS-BvN-D7, as well as more types of lectins in survivors from breeding strains. Hence, in survivors of the breeding strain, the complement components reported in the acute phase in common carp during CyHV-3 infection (53), could facilitate the phagocytic process *via* binding of MRC (Mannose-Receptor C) in fish (54). The interleukin 21 receptor (IL21R), which was found regulated in the survivors of the breeding strain for group SvC-B and the healthy control of the non-breeding strain for group SvC-N, indicated the control of inflammation by suppressing STAT3 signaling (55). The upregulated IL23R in the survivors of the breeding strain for group SvC-B can promote cytotoxic ability (56), which may immediately kill infected cells. The lack of antigen presentation in comparison group SvC-B suggested that the infection was overcome before the amplification of CyHV-3 in the breeding strain. As to the BS-advantage interferon related genes in group SvS-BvN-D7, the activator of IRF7 (57) as well as MX, the inhibitor of virus replication, may elicit the interferon antiviral response. Meanwhile, for proinflammatory cytokine, echoed with upregulated IL-1, IL-6, and IL-8 in group SvC-N, the NBS-advantage proinflammatory signaling dominated in the survivors from non-breeding strain, and also numerous upregulated genes were involved in antigen presentation in the non-breeding strain in group SvC-N. Therefore, the survivors from breeding strains overcame the infection mainly through phagocytosis and cytotoxicity at the cellular level and may elicit IFN response, without activating the typical process of proinflammation as in survivors from the non-breeding strain.

Thus, there are different signatures between the two strains, regarding the survival strategy. For the survivors in the breeding strain, self-repairing related autophagy was detected as both the KEGG pathways “regulation of autophagy” and autophagy-related fish antiviral tripartite motif (TRIM) protein (58) were found in group SvC-B. These findings were in line with TRIMs, which were found regulated in the survivors of breeding strain for group SvC-B and in the healthy control of non-breeding strain for group SvC-N. The above finding of TRIM is coincident with one of the recently revealed CyHV-3 resistant related DEGs with identified QTLs (17). In the survivors from the breeding strain, the higher PI3K also suggests higher autophagy, since the PI3K/AKT/mTOR pathway enhances this process (59). In the breeding strain, the suppressed IFN activation was also suggested, for that fish TRIM may inhibit the activation of IFN and attenuate IFN regulatory factor (IRF) (60, 61). The factor that there was more expression of TRAF6 in survivors of breeding strain compared with those of non-breeding strain, is in line with the resistant related SNP on TRAF6 (62) and suggests the possible repression on the production of type I IFN (63). In addition, nuclear factor, interleukin 3 regulated (NFIL3) can control Treg cell function *via* directly binding to and negatively regulates the expression of Foxp3 (64), and has been revealed stimulating both proinflammatory (e.g., NF-kappa B [NF-κB]) and anti-inflammatory factors (e.g., IL10) in carp (65). Thus, the upregulated NFIL3 in the survivors of the breeding strain for group SvC-B may suggest the extensive activation of immune cells, with diminished immune regulation. The directed lymphocytes response could be present as there was upregulation of IL23R, which significantly enhances the expression of cytotoxic mediators (56), as well as cathepsin L (a component of lysosomes) (66). This indicated an enhanced activation of the cytotoxic ability of T cells in survivors from the breeding strain. For B cells, the secretion of Ig-related genes (e.g., polymeric immunoglobulin receptor [PIGR], Ig heavy chain and light chain) was also upregulated in the survivors of breeding strain for group SvC-B. To fight the virus, the survivors from the non-breeding strain developed typical inflammatory cascades, including pro-inflammatory cytokines (e.g., IL-1, IL-6, and IL-8), as well as MAPK signaling, which is profoundly involved in cell survival functions during viral infection (67). For the control of inflammation, the survivors from the common strain exhibited suppression of the IFN response, which was also reflected by the upregulated IRF1 and IRF8, the inhibitor of the MYD88-mediated NF-κB signaling pathway (68, 69). Downstream hypoxia was also found, as indicated by the “p53 signaling pathway” and “oxidative phosphorylation”. The hypoxic status could also protect survivors of the non-breeding strain from death since p53 suppresses cell apoptosis (70), and HIF1A regulates virus-induced glycolytic genes (71).

Furthermore, the DEGs generated by comparing the survivors from the two strains in comparison group SvS-BvN-D7, which can provide clues for how to develop the resistance to CyHV-3 infection. The BS-advantage semaphorin, which was also regulated by LncRNA, is related to immunoregulation (72, 73). This indicated a possible tolerance of viral replication or latency after primary infection in the survivors (2), while had no negative effects on the proliferation of host cells (74). There was less cyclin-dependent kinase inhibitor 1D (CDKN1D) in survivors from the breeding strain compared with the non-breeding strain. This indicated that there was an interrupted circadian cell-cycle timing in the survivors from non-breeding strains (75). The higher levels of STAT3 and possible its upregulated integrins (76) in survivors from the non-breeding strain may facilitate the effect of enhanced IL6 signaling. Apart from the typical immune signals in comparison group SvS-BvN-D7, there was relatively higher PI3K activity, which was involved in the KEGG pathway “dopaminergic synapse”. This indicated that dopamine inhibited inflammation (77) in survivors from the breeding strain. This is because PI3K is dependent on the accumulation of DOPA decarboxylase, the enzyme involved in the production of dopamine, which is reduced by a viral infection (78). Additionally, genes responsive to the secretion of immunoglobulin (e.g., PIGR and Ig light chain), which is important for anti-virus immunity, were common (in comparison group SvI-B&N) between the two strains.

Additionally, the comparison of two strains with health status in group CvC-BvN-D0 suggested the differential basal properties for immune homeostasis. Among BS-advantage immune DEGs, which was related to the healthy control of breeding strain, the expression of NACHT, LRR, and PYD domains-containing protein 3 (NALP3) indicated a stronger ability to form the inflammasome in the breeding strain. The higher levels of NLR family CARD domain containing 3 (NLRC3), which is a negative regulator of the DNA sensor STING, suggest less sensitivity to the DNA virus in the breeding strain. The higher levels of MHC I indicate greater potential to activate response by cytotoxic CD8^+^ T cells upon herpes virus infection, as previously revealed in the resistant strain R3 (79). For the potential T helper cell differentiation-related JAK/STAT pathway, the upregulation of both STAT3 and histone deacetylase (HDAC) indicated an easier differentiation of both T helper 17 and regulatory T directions (80), respectively, in the breeding strain even in the steady state (81, 82). Upregulation of HDAC in the healthy control of breeding strain for group CvC-BvN-D0, which could inhibit the function of macrophages in fish (83), also suggests a more delicate immune regulation during the steady state in the breeding strain. The macrophage related MRC (Mannose-Receptor C) in fish could potentially induce the phagocytic process if necessary (54). However, among NBS-advantage immune DEGs, which indicated higher expression in the non-breeding stain at a steady state, myeloid-associated differentiation marker-like protein 2 (myadml2) suggested the basic more differentiation of monocyte cell types (84) As the surface receptor, TLR18 and ITGB1 suggested susceptibility to virus binding. Moreover, C-C motif chemokine ligand 4 (CCL4), which can protect infected cells with the viral burden (85), may be a risk factor for the non-breeding strain. In addition, the downregulated DEGs in group SvC-B and SvC-N also provided clues for how to maintain immune homeostasis, which can prevent the virus challenge. Among the downregulated DEGs in group SvC-B, both the existence of C3 and IFI suggested a restricting effector on the virus, since that C3b can bind directly to purified glycoprotein C of herpesvirus (86), and interferon-induced protein 44 (IFI44) may restrict virus replication (87). These factors were also revealed in NBS-advantage immune DEGs in group CvC-BvN-D0. Therefore, upon steady state, C3b and IFI44 also served as the basic defense function even in the virus carrier state, which could be especially important for the non-breeding strain.

The revealed regulation of resistance to CyHV-3 by lncRNA involved numerous biological processes. Among the revealed GO terms in comparison group SvC-N, “DNA replication” could indicate virus proliferation in the non-breeding strain. In survivors from the non-breeding strain, the lncRNA-regulated genes were mainly involved in innate immune cell function (e.g., macrophage protein and cathepsin, as components of lysosomes) and cell apoptosis. Nramp and PAI were found regulated by LncRNA in comparison groups SvC-N (up) and SvS-BvN-D7 (down). This finding indicates the more expression of Nramp and PAI in the survivors of non-breeding strain, could facilitate virus infection and proliferation for infected cell respectively, since that Nramp may serve as a virus receptor (88), meanwhile, PAI can inhibit apoptosis in cell lines infected with viruses (89). While, in survivors from the breeding strain, the only lncRNA-modulated gene, TLX3, is strongly methylated. This indicates that TLX3 expression was suppressed during hepatitis B virus-related cancer (90). This suggests that TLX3 in survivors from the breeding strain may play a protective role, and participate in lymphocyte proliferation in the head kidney. Meanwhile, echoing with the recent finding of the participation of zebrafish galectin proteins in immunity against viral infection (48, 91), in comparison group SvS-BvN-D7 the LncRNA regulated transcripts Lgals3l (down) and Lgals3bpa (up), as well as BS-advantage Lgals3bpa and galectin 3 suggested the regulation of galectin-3 related biological activities could be related to reduce the viral attachment for survivors of the breeding strain.

**Figure 7:**
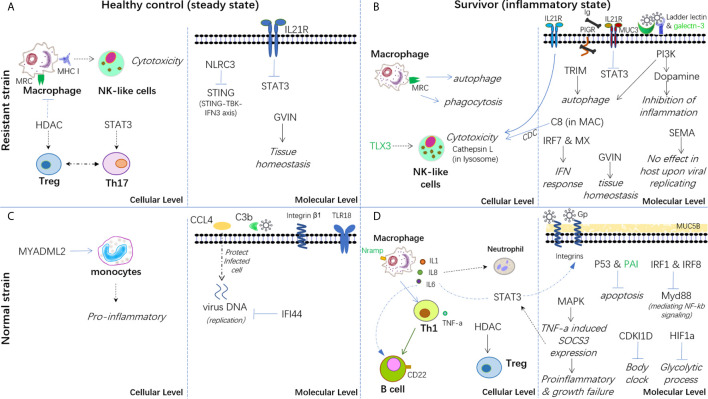
Hypothesis of the carp immune mechanism in both breeding (resistant) and non-breeding (normal) strains, either at the steady-state or upon surviving for CyHV-3 infection. **(A)** Healthy control in the steady state in the breeding strain. **(B)** Survivors in an inflammatory state in the breeding strain. **(C)** Healthy control in the steady state in non-breeding strain. **(D)** Survivors in an inflammatory state in the non-breeding strain. The words in italics, bold, and green represent the biological process, cell type, and lncRNA-modulated immune genes, respectively. The arrow denotes facilitation or promotion, and --- denotes inhibition. The dashed line indicates a possible correlation.

Therefore, based on the present findings, a hypothesis has been proposed for the immune mechanisms involved in both healthy controls and survivors from infection in both strains (**Figure 7**). In conclusion, the breeding strain of common carp showed a better ability to maintain immune homeostasis in both steady and inflammatory states and displayed enhanced blockage of CyHV-3 infection compared with the non-breeding strain. Thus, this strain could be termed as a resistant strain accordingly. Since the modulation of mRNA and lncRNA expression dynamics currently remains a hypothesis, further molecular evidence is needed to elucidate the mechanism of both resistance and susceptibility. In addition, the finding that both the inhibition of inflammation by dopamine in the breeding strain and the disrupted bio-clock in the non-breeding strain upon CyHV-3 infection suggested better growth performance for the breeding strain. Therefore, the possible advantage of this resistant strain for growth performance warrants further study. 

## Data Availability Statement

The datasets presented in this study can be found in online repositories. The names of the repository/repositories and accession number(s) can be found in the article/**Supplementary Material**.

## Ethics Statement

The animal study was reviewed and approved by The Committee for the Welfare and Ethics of Laboratory Animals, Heilongjiang River Fisheries Research Institute, Chinese Academy of Fishery Sciences.

## Author Contributions

LS and ZJ conceived the project and designed the experiments. NW and ZJ wrote the manuscript. ZJ performed the experiments. ZJ and JXS conducted the CyHV-3 infection experiment and collected samples. CL, XH, and YG performed fish propagation and culture. XJ conducted the RT-PCR experiment. NW and X-QX coordinated the data analysis tasks. NW, HL, MS, WY, YT, JWS, and YC analyzed the data. The manuscript was revised and approved by X-QX and LS. All authors contributed to the article and approved the submitted version.

## Funding

This work was funded by grants from the National Key R&D Program of China (2018YFD0900302-6), The Natural Science Foundation of Heilongjiang Province (TD2019C004), China Aquaculture Research System (CARS-45-06), and Central Public-interest Scientific Institutional Basal Research Fund (2020TD31).

## Conflict of Interest

The authors declare that the research was conducted in the absence of any commercial or financial relationships that could be construed as a potential conflict of interest.
