# Supplementary material for: Integrative Transcriptomic Analysis Reveals the Immune Mechanism for a CyHV-3-Resistant Common Carp Strain
Source: Front Immunol. 2021 Jul 5;12:687151. doi: 10.3389/fimmu.2021.687151 (PMC8287582; doi:10.3389/fimmu.2021.687151)
Supplement: Supplementary file 1 [file DataSheet_1.pdf]

A: the black module

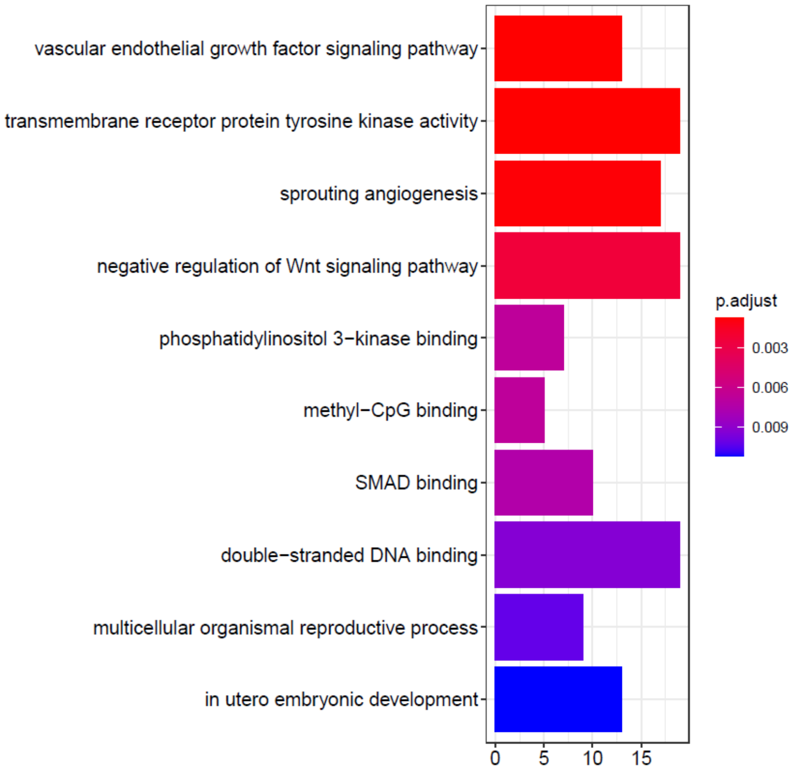

B: the blue module

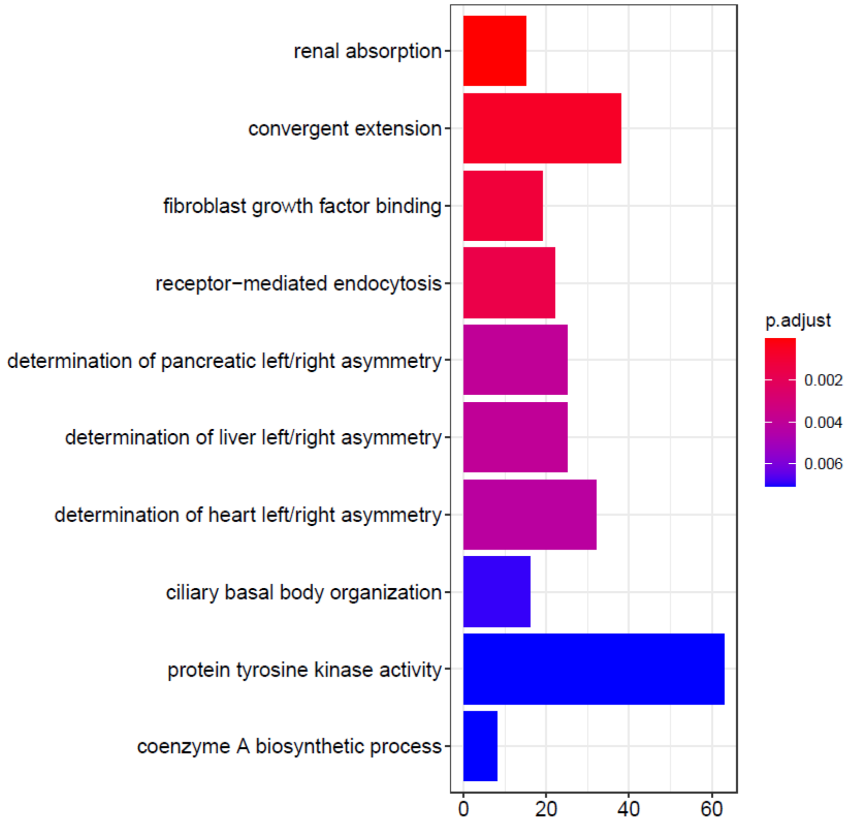

C: the brown module

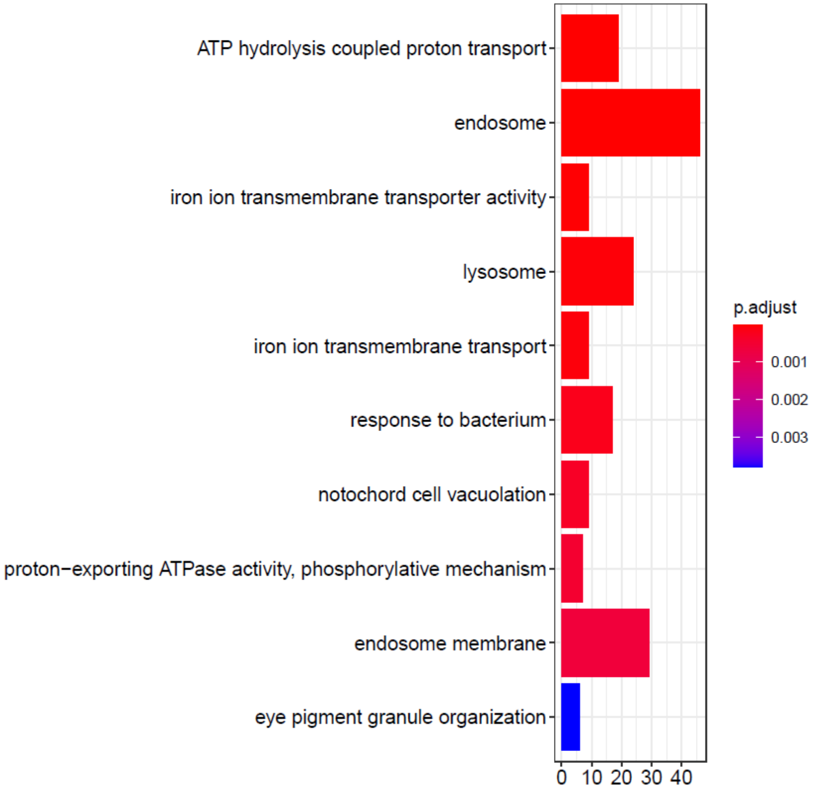

D: the dark orange module

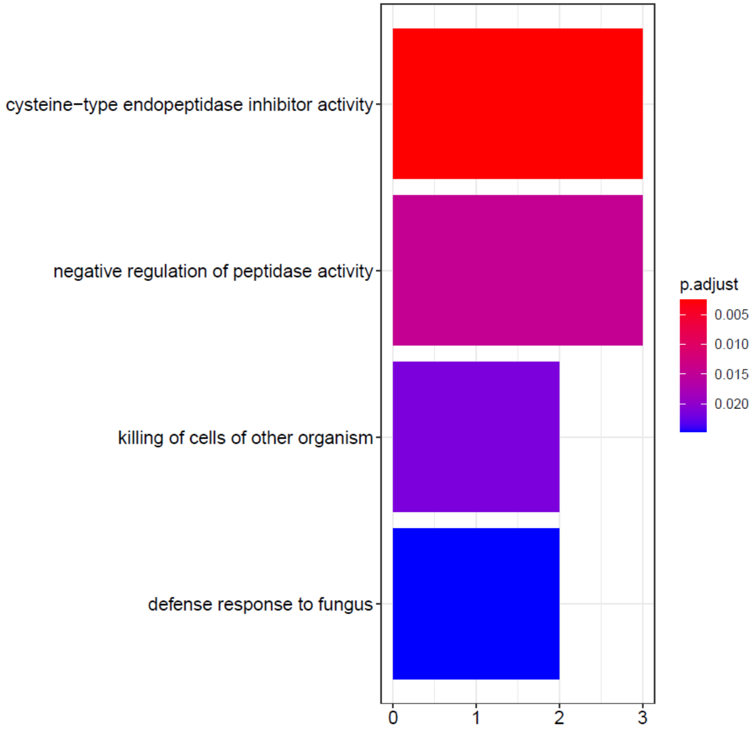

E: the grey 60 module

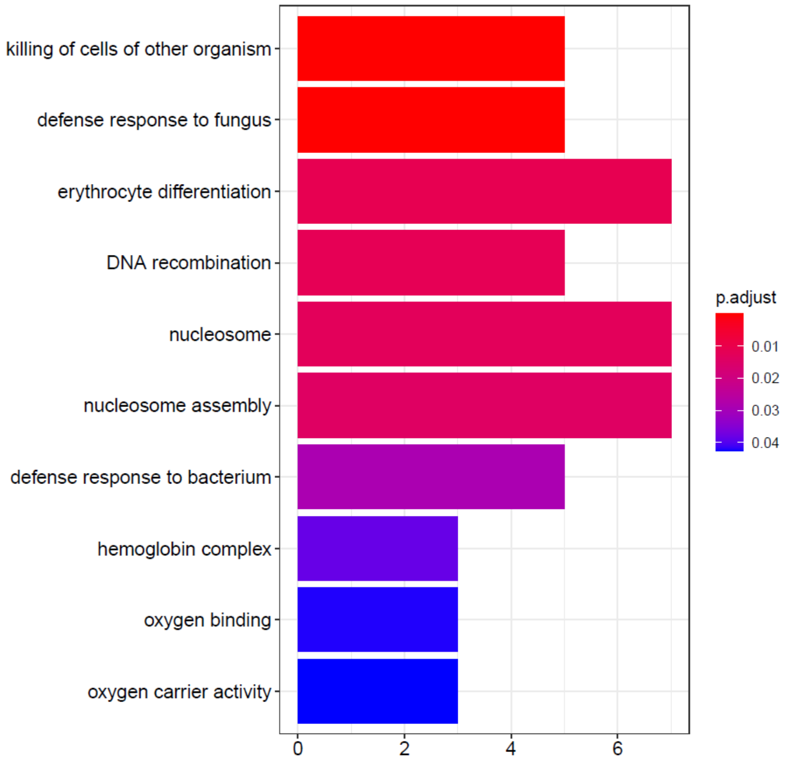

F: the light green module

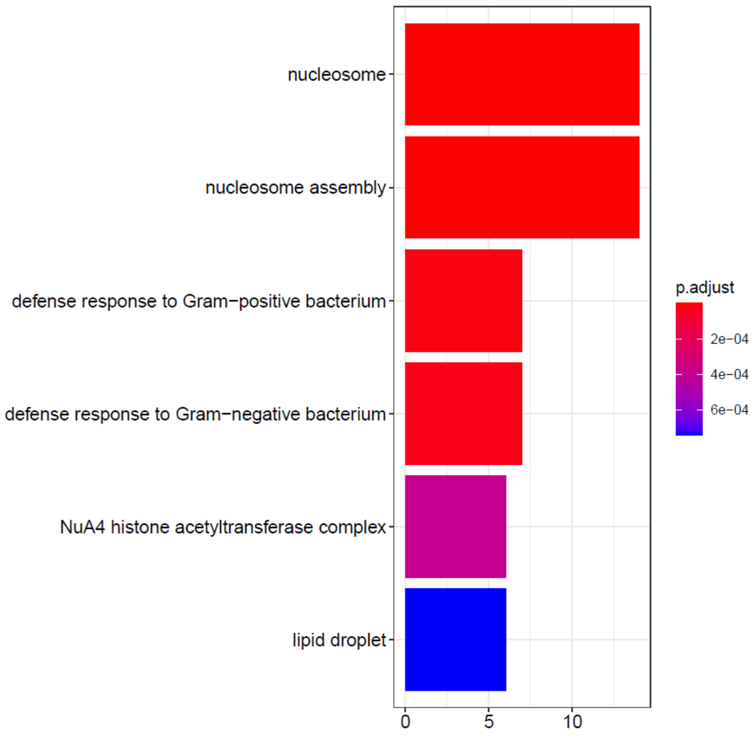

G: the light yellow module

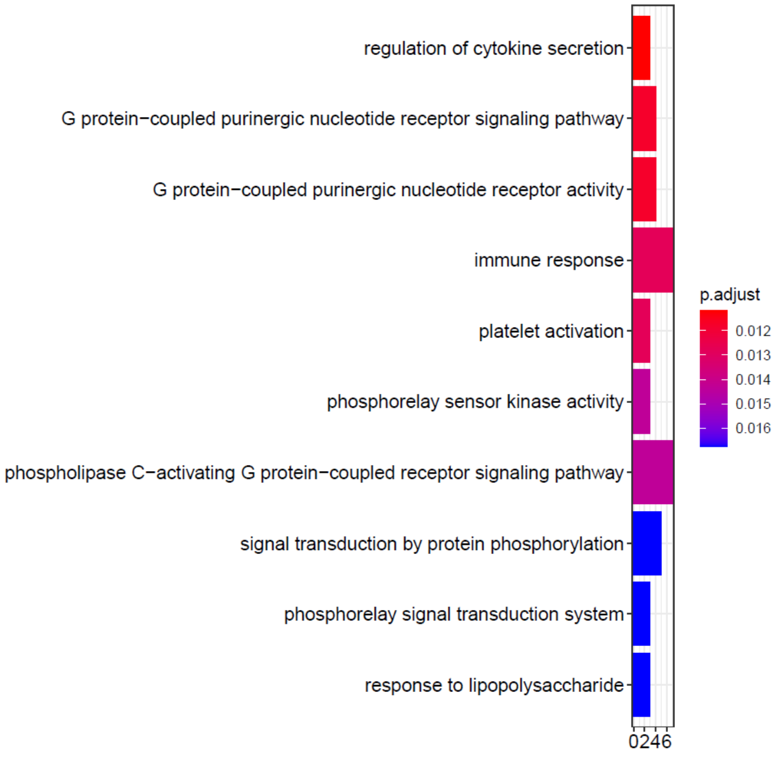

H: the midnight blue module

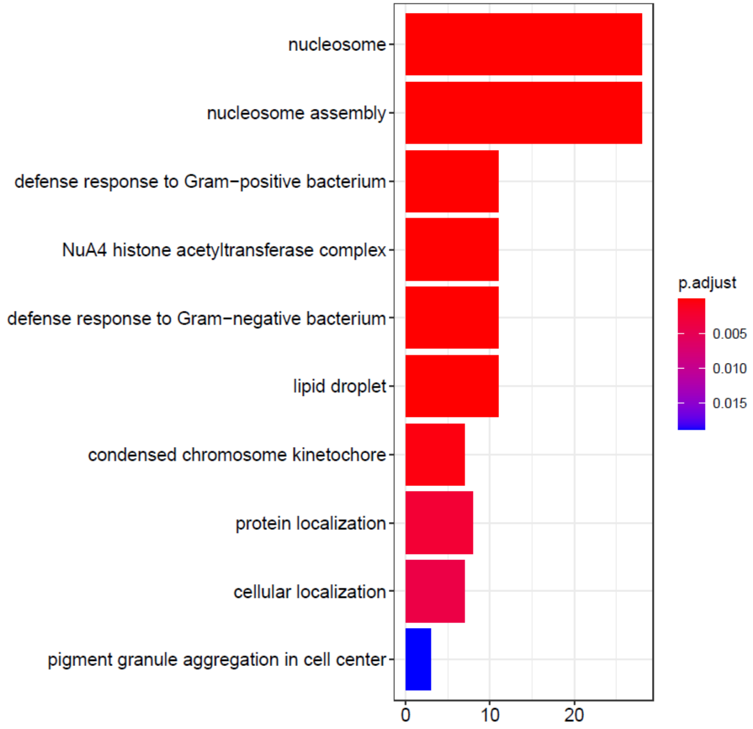

I: the orange module

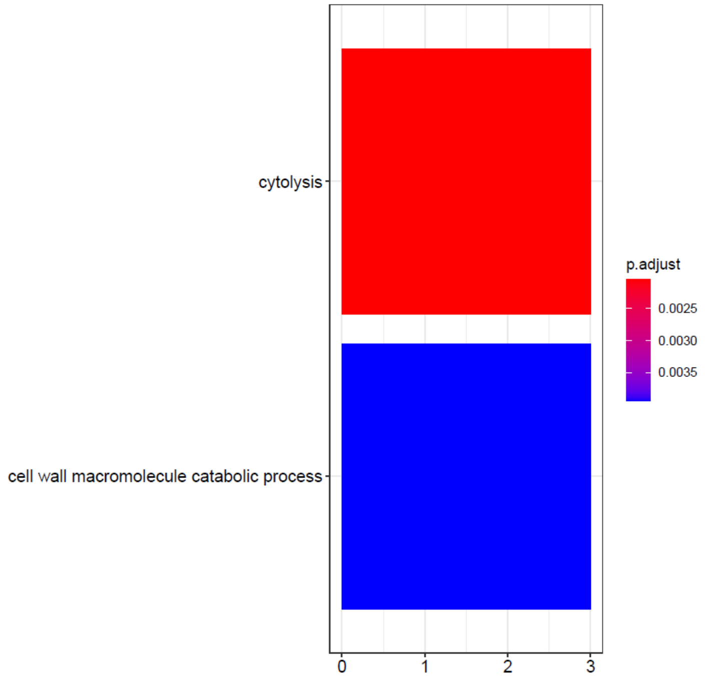

J: the pink module

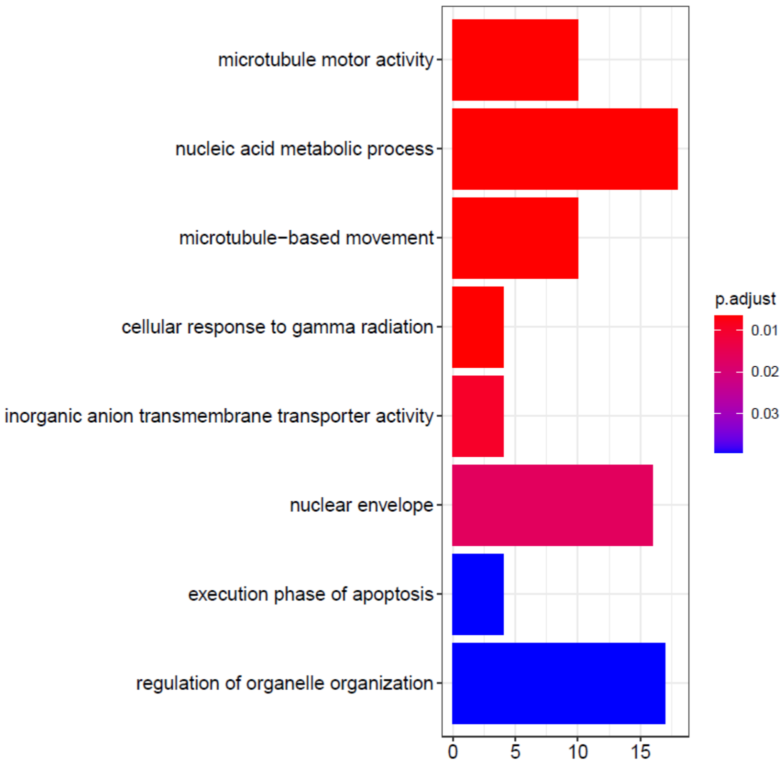

K: the red module

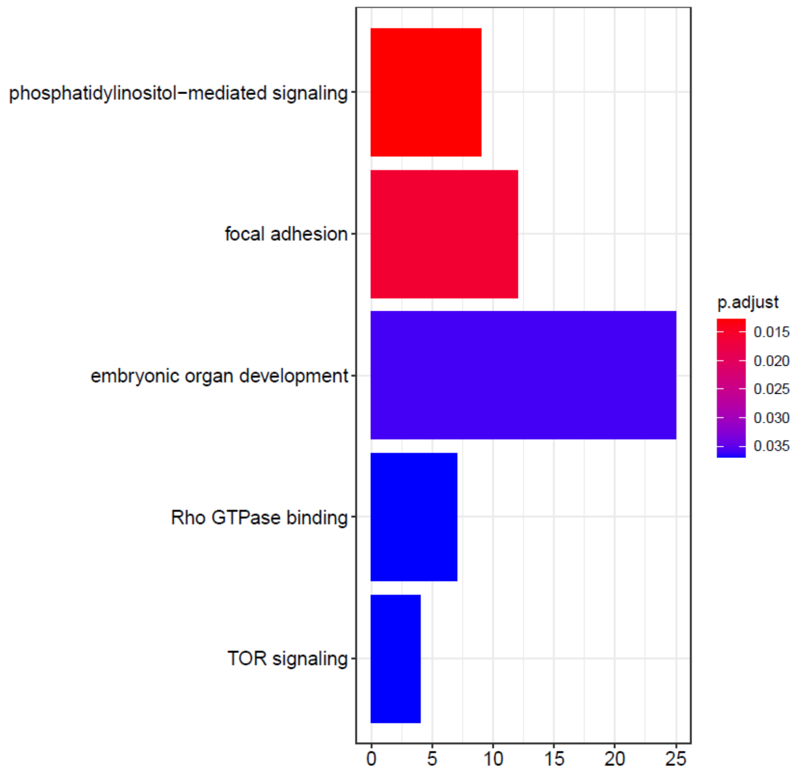

L: the turquoise module

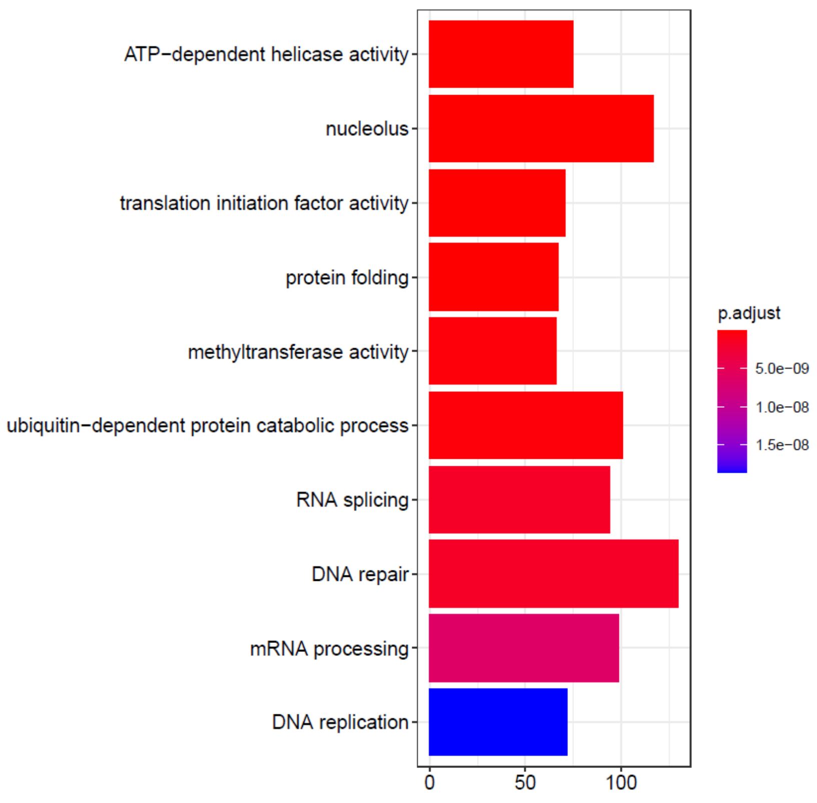

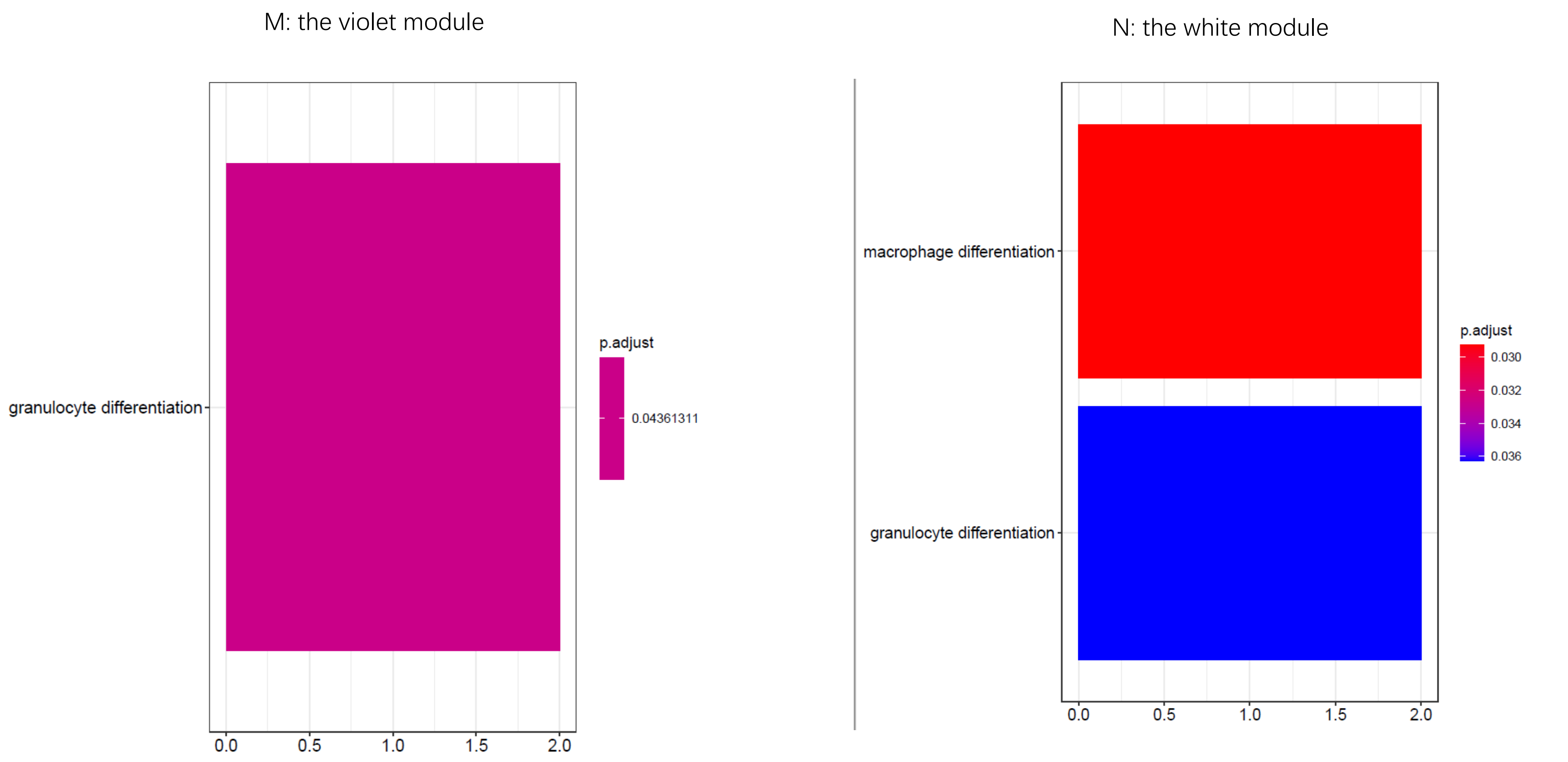

Fig. S1 the immune GO terms containing module
